# Arsenic species and lipid metabolism under low-to-moderate arsenic exposure: evidence from NHANES and a Chinese adult population

**DOI:** 10.3389/fendo.2026.1919240

**Published:** 2026-08-18

**Authors:** Di Wu, Shirui Yan, Xinxiao Li, Jing Wang, Nian Gao, Minghan Luo, Xiaowei Wang, Ailin Li, Rui Zhang, Lei Wu, Junrui Pei

**Affiliations:** 1 Key Lab of Etiology and Epidemiology, Education Bureau of Heilongjiang Province & Ministry of Health, Center for Endemic Disease Control, Chinese Center for Disease Control and Prevention, Harbin Medical University, Harbin, Heilongjiang, China; 2 Public Health College, Qiqihar Medical University, Qiqihar, Heilongjiang, China

**Keywords:** arsenic species, lipid metabolism, mediation analysis, urinary albumin-to-creatinine ratio, urinary arsenic

## Abstract

**Background:**

The relationship between low-to-moderate arsenic exposure and lipid metabolism remains unclear, particularly for different urinary arsenic species and the potential role of urinary albumin-to-creatinine ratio (UACR).

**Methods:**

This cross-sectional study used NHANES 2009–2020 data and an adult population from Zhaodong, China. The NHANES analysis included 2,104 participants, representing 52,612,536 adults, and the Zhaodong analysis included 310 participants. Creatinine-adjusted urinary total arsenic (UTA) and dimethylarsinic acid (DMA) were designated as the principal exposure indicators, whereas monomethylarsonic acid (MMA), arsenite (As^III^), and arsenate (As^V^) were evaluated in supplementary exploratory analyses. Triglyceride (TG), low-density lipoprotein cholesterol (LDL-C), non-high-density lipoprotein cholesterol (non-HDL-C), and UACR were examined as outcomes. Multivariable regression, restricted cubic spline, subgroup, and mediation analyses were performed.

**Results:**

The median UTA concentrations were 3.73 μg/L in NHANES and 12.37 μg/L in the Zhaodong population. In NHANES, after multivariable adjustment, UTA and DMA were positively associated with LDL-C levels, with β values of 0.68 and 0.85, respectively, and both associations remained significant after FDR correction (*q* < 0.1). Restricted cubic spline analyses revealed a nonlinear association between DMA and LDL-C. In the Zhaodong population, the highest quartile of DMA was associated with lower odds of abnormal LDL-C compared with the lowest quartile. In NHANES, both UTA and DMA were positively associated with UACR. Mediation analyses did not support a meaningful mediating role of UACR, although small negative indirect effects were observed in the LDL-C models involving UTA and DMA. Supplementary exploratory analyses involving MMA, As^III^, and As^V^ yielded several nominal associations.

**Conclusions:**

Low-to-moderate urinary arsenic exposure may be associated with altered LDL-C levels and increased UACR, with species- and population-specific differences. The mediation analyses did not support a meaningful mediating role of UACR. Prospective studies are needed to confirm these findings.

## 1 Introduction

Environmental and dietary sources, particularly contaminated drinking water, food, and occupational settings, are major contributors to human arsenic exposure (1). After entering the body, inorganic arsenic is metabolized into several urinary species, mainly arsenite (As^III^), arsenate (As^V^), monomethylarsonic acid (MMA), and dimethylarsinic acid (DMA) (2). DMA usually represents the largest fraction of urinary arsenic and is often used to reflect arsenic methylation capacity (3). However, urinary total arsenic (UTA) alone may mask heterogeneity in arsenic biotransformation because individual species differ in metabolism, biological activity, and toxicity. Accordingly, species-specific urinary arsenic profiles may provide additional insight into arsenic exposure patterns and their potential health relevance.

Although arsenic is best known for its carcinogenicity, its potential effects on cardiovascular and metabolic health have received increasing attention (4). Large populations worldwide remain exposed to arsenic at concentrations that may threaten human health, underscoring its continuing importance as an environmental health issue (5). In recent years, increasing attention has been given to the cardiometabolic effects of arsenic. Experimental and epidemiological studies suggest that arsenic exposure may impair vascular and metabolic homeostasis through oxidative stress, inflammatory activation, endothelial injury, and altered lipid regulation (6, 7).

Abnormal lipid metabolism is central to the development of atherosclerotic cardiovascular disease (ASCVD) (8). Among commonly used lipid indicators, low-density lipoprotein cholesterol (LDL-C) represents a key therapeutic target, non-high-density lipoprotein cholesterol (non-HDL-C) reflects the total atherogenic lipoprotein burden, and triglycerides (TG) reflect triglyceride-rich lipoprotein metabolism and residual cardiovascular risk (9). Although dyslipidemia is strongly influenced by lifestyle, adiposity, metabolic status, and genetic background, environmental exposures are increasingly being considered as additional contributors to adverse lipid profiles and cardiovascular risk (10).

Epidemiological evidence suggests that arsenic exposure may alter lipid metabolism (11). In populations with higher or sustained exposure, arsenic has been positively associated with atherogenic lipid indicators, including LDL-C, TG, and non-HDL-C, and inversely associated with HDL-C (11, 12). However, evidence at moderate exposure levels remains inconsistent. One study conducted under moderate drinking-water arsenic exposure conditions (<150 μg/L) reported a positive association between UTA and HDL-C, suggesting that lipid association patterns may differ from those observed at higher exposure levels (13). Another adult study linked higher urinary total arsenic and selected species, including DMA and MMA, to abnormal LDL-C (14). A recent systematic review indicated that associations between low-to-moderate drinking-water arsenic exposure (drinking-water arsenic <100 μg/L) and cardiovascular outcomes remain heterogeneous across populations, and no consistent conclusion has been reached (15). Given that lipid metabolic abnormalities may serve as early metabolic phenotypes of cardiovascular risk, evidence regarding lipid metabolic changes under low-to-moderate arsenic exposure conditions is even more limited, particularly regarding whether different urinary arsenic species are differentially associated with LDL-C, non-HDL-C, and TG.

Urinary albumin-to-creatinine ratio (UACR) reflects urinary albumin excretion and is commonly used to identify early glomerular injury. Small increases in UACR, even within the conventional normal range, have been associated with higher cardiovascular risk and may indicate systemic microvascular or endothelial dysfunction (16, 17). UACR has also been linked to lipid-related abnormalities, particularly higher TG and total cholesterol levels and lower high-density lipoprotein cholesterol concentrations (18–20). In kidney disease or overt proteinuria, urinary protein loss may disturb lipid metabolism by affecting lipoprotein synthesis, clearance, and lipolytic activity (21). Because arsenic exposure has also been associated with albuminuria, increased UACR, and renal dysfunction markers (22–24), UACR may represent a potential pathway connecting urinary arsenic exposure with lipid metabolic alterations. However, this potential role has not been well characterized in population-based studies.

To address these gaps, we analyzed data from NHANES 2009–2020 to examine the associations of urinary arsenic biomarkers with LDL-C, non-HDL-C, and TG among adults with low-to-moderate arsenic exposure, with primary emphasis on UTA and DMA. Because of the relatively high proportions of measurements below the LOD, analyses involving MMA, As^III^, and As^V^ were considered supplementary and exploratory. We also examined the associations of urinary arsenic biomarkers with UACR and explored whether UACR was involved in arsenic–lipid relationships. A comparative analysis was subsequently conducted among adults from Zhaodong, Heilongjiang Province, China, a non-endemic arsenic exposure area, to assess the consistency and heterogeneity of the observed associations across different population settings. Overall, this study aimed to characterize the relationships between low-to-moderate urinary arsenic exposure and lipid metabolism and to generate further evidence regarding species-specific patterns and the possible involvement of UACR.

## 2 Materials and methods

### 2.1 NHANES analysis

#### 2.1.1 Inclusion and exclusion criteria

Data were drawn from five NHANES cycles conducted between 2009 and 2020 (25). All NHANES participants provided written informed consent, and the survey protocols were approved by the NCHS Research Ethics Review Board. Urinary arsenic concentrations were analyzed as biomarkers of internal exposure to investigate their associations with lipid metabolism indicators and UACR under low-to-moderate environmental exposure conditions (26). The initial dataset included 55,999 participants. We first excluded participants who were pregnant, refused to answer, or responded “don’t know” regarding pregnancy status (n = 329), as well as those aged <20 years (n = 23,501), leaving 32,169 non-pregnant adult participants. Participants with missing data on key variables were then excluded, resulting in 4,727 individuals with complete baseline information. Notably, urinary total and speciated arsenic biomarkers were not measured in the full NHANES sample but in a predefined one-third laboratory subsample of participants aged ≥6 years. Therefore, much of the reduction at this stage reflected the NHANES laboratory subsampling design rather than investigator-defined exclusion. We further excluded participants who were using lipid-lowering medications (n = 452), because these medications directly modify serum lipid concentrations and could therefore obscure the associations between urinary arsenic biomarkers and lipid outcomes. Participants with urinary arsenobetaine (AsB) concentrations >1.20 μg/L (n = 2,171) were excluded to limit the influence of seafood-derived organic arsenic on exposure assessment. This threshold corresponded approximately to the highest lower limit of detection for urinary AsB across the NHANES 2009–2020 survey cycles. The final main analytic sample included 2,104 participants. Because of missing data for different lipid metabolism indicators, outcome-specific analytic samples were constructed for TG (n = 959), LDL-C (n = 950), and non-HDL-C (n = 2,104) (**Supplementary Figure 1**).

#### 2.1.2 Urinary arsenic measurements

Low-to-moderate arsenic exposure was defined according to previous studies as drinking water arsenic concentrations below 100 μg/L or urinary concentrations of inorganic arsenic species and their methylated metabolites within ranges typically observed in environmentally exposed populations (15, 26, 27). Urinary arsenic concentrations are generally used to characterize individual internal exposure rather than as specific cutoffs for classifying low, moderate, or high arsenic exposure. Therefore, the term “low-to-moderate exposure” does not imply that different study populations have identical or similar urinary arsenic concentrations or exposure levels. UTA and individual arsenic species, including As^III^, As^V^, MMA, DMA, and AsB, were measured using inductively coupled plasma mass spectrometry (ICP-MS). Measurements below the limit of detection (LOD) were assigned a value of LOD/√2. To reduce variability due to spot urine dilution, urinary arsenic concentrations were normalized by urinary creatinine and reported as μg/g creatinine (28). To minimize interference from seafood-derived organic arsenic related to recent seafood intake, only participants with urinary AsB concentrations ≤1.20 μg/L were included (29). The LODs, sample sizes, and detection rates of urinary arsenic across survey cycles are presented in **Supplementary Table 1**. All laboratory measurements were performed according to NHANES laboratory standard operating procedures.

#### 2.1.3 Measurements of lipid metabolism indicators and UACR

The primary lipid metabolism indicators were TG, LDL-C, and non-HDL-C, while UACR was examined as a potential mediator linking urinary arsenic exposure to lipid metabolism. TG was assessed enzymatically, whereas HDL-C was measured after precipitation of apolipoprotein B-containing lipoproteins. LDL-C was estimated using the Friedewald formula: LDL-C = total cholesterol (TC) − HDL-C − TG/5, with all lipid values expressed in mg/dL. When TG concentrations exceeded the applicable range of the Friedewald equation, the corresponding LDL-C values were coded as missing by NHANES. Non-HDL-C was derived by subtracting HDL-C from TC. UACR was calculated as the ratio of urinary albumin to urinary creatinine and expressed as mg/g. Urinary albumin was measured by fluorescent immunoassay, and urinary creatinine was assessed using a Beckman Synchron CX3 clinical analyzer.

#### 2.1.4 Covariates

Variables considered as potential confounders included age, sex, race/ethnicity, education level, poverty income ratio (PIR), serum cotinine, hypertension status, body mass index (BMI), estimated glomerular filtration rate (eGFR), urinary AsB, diabetes status, and survey cycle. PIR was calculated as annual household income divided by the poverty threshold and is commonly used in NHANES as an indicator of socioeconomic status. PIR was categorized as ≤1.3, 1.3–3.5, and >3.5 (30). Serum cotinine, the major metabolite of nicotine, was used as an objective biomarker of active smoking and secondhand smoke exposure and was categorized as <0.05, 0.05–10, and >10 ng/mL, representing no or minimal tobacco smoke exposure, secondhand or low-level tobacco smoke exposure, and higher-level tobacco exposure, respectively (31). eGFR was calculated using the established equation (32). Hypertension was defined as the average of three systolic blood pressure measurements ≥140 mmHg, the average of three diastolic blood pressure measurements ≥90 mmHg, self-reported current use of antihypertensive medication, or self-reported physician-diagnosed hypertension. Urinary AsB was included to control for the potential influence of seafood-derived organic arsenic exposure. Diabetes was defined as self-reported physician-diagnosed diabetes or current use of glucose-lowering medication. Urinary creatinine standardization was used to account for variations in spot-urine dilution, whereas eGFR, which was primarily derived from serum creatinine, was included to account for kidney function that may influence urinary arsenic excretion and lipid metabolism, consistent with previous epidemiological studies (22, 27).

### 2.2 Zhaodong population analysis

#### 2.2.1 Study population and inclusion/exclusion criteria

The comparative Chinese sample was recruited from Zhaodong City, Heilongjiang Province, China, an area without a recognized history of endemic arsenic poisoning. Given China’s current drinking-water arsenic standard of 10 μg/L, this population was considered representative of a low-to-moderate exposure setting for comparative analysis (15). A cross-sectional survey was conducted in November 2024, and 488 adults were initially enrolled. Ethical approval was granted by the Ethics Committee of the Endemic Disease Control Center at Harbin Medical University, and all participants gave written informed consent. After excluding participants with missing smoking status, TG, or urinary creatinine data, 417 individuals remained. Participants with missing UTA or arsenic species data, as well as those with extreme UTA values, were further excluded. The final Zhaodong analytic sample included 310 adults. The participant selection flowchart is shown in **Supplementary Figure 2**.

#### 2.2.2 Urinary arsenic measurements

UTA was determined by hydride generation–atomic fluorescence spectrometry (HG-AFS). Urinary arsenic species, including As^III^, As^V^, MMA, DMA, and AsB, were analyzed by high-performance liquid chromatography coupled with hydride generation–atomic fluorescence spectrometry (HPLC-HG-AFS). Species separation was performed using a PRP-X100 anion-exchange column (Hamilton, Switzerland). Ultrapure water adjusted to pH 9.0 and 50 mmol/L diammonium hydrogen phosphate solution adjusted to pH 6.0 were used as mobile phases, and elution was conducted according to a predefined gradient program. Detection was performed using an AFS-933 atomic fluorescence spectrometer (Beijing Jitian Instruments Co., Ltd., China). Arsenic species standards were obtained from the National Institute of Metrology, China, and concentrations were quantified using an external standard method. Blank and quality control samples were included in each analytical batch. Measurements below the LOD were assigned a value of LOD/√2, and the corresponding LODs are presented in **Supplementary Table 1**.

#### 2.2.3 Measurements of lipid metabolism indicators and UACR

TG, TC, HDL-C, and LDL-C concentrations were measured in batches using an automated biochemical analyzer (Hitachi Ltd., Japan). UACR was derived as the ratio of urinary albumin to urinary creatinine.

#### 2.2.4 Covariates

Potential confounders included age, sex, race/ethnicity, education level, annual household income, BMI, eGFR, smoking status, hypertension status, and frequency of aquatic product or seafood consumption. Annual household income was classified into three categories: <20,000–60,000, and >60,000 Chinese yuan. BMI and eGFR were analyzed as continuous variables. Smoking status was categorized as never, former, or current smoking. Hypertension was defined using the same criteria as in the NHANES analysis. Frequency of aquatic product or seafood consumption was categorized as no consumption, at least once per day, at least once per week, at least once per month, and at least once per year.

### 2.3 Statistical analysis

For NHANES, we incorporated survey weights to account for the complex sampling design. Baseline characteristics, urinary arsenic exposure levels, and lipid metabolism indicators were summarized using descriptive statistics. Urinary arsenic exposure was assessed using UTA and individual urinary arsenic species, including As^III^, As^V^, MMA, and DMA. UTA and DMA were treated as the principal exposure indicators. Given the relatively high proportions of measurements below the LOD for MMA, As^III^, and As^V^, analyses involving these species were designated as supplementary exploratory analyses and were not used as the primary basis for inference. Participants were divided into quartiles according to each urinary arsenic indicator. Multivariable linear regression models were fitted to examine associations of urinary arsenic exposure with continuous lipid metabolism indicators and UACR. Multivariable logistic regression models were used to estimate associations between urinary arsenic exposure and lipid abnormalities, and trend tests were performed across quartiles. To improve comparability between NHANES and the Zhaodong population, the primary multivariable models preferentially included covariates that were available and similarly defined in both datasets. NHANES models were adjusted for age, sex, race/ethnicity, education level, PIR, serum cotinine, hypertension status, BMI, eGFR, urinary AsB, and survey cycle. Zhaodong models were adjusted for age, sex, ethnicity, education level, annual household income, smoking status, hypertension status, BMI, eGFR, and frequency of aquatic product or seafood consumption.

Restricted cubic spline (RCS) models were applied to describe the exposure–response patterns between urinary arsenic indicators and lipid metabolism outcomes. Subgroup analyses were performed separately for NHANES and the Zhaodong population to examine whether these associations varied by participant characteristics. Effect modification was evaluated by adding interaction terms between urinary arsenic indicators and stratification variables to the regression models. Stratified analyses were conducted by age, sex, BMI, and hypertension status in both populations. BMI was grouped as non-overweight (<25 kg/m²), overweight (25–30 kg/m²), or obese (≥30 kg/m²) (33). Tobacco-related stratification was based on serum cotinine in NHANES and smoking status in the Zhaodong population. The subgroup models used the same covariate adjustment strategy as the main models, except that the corresponding stratification variable was not included. Mediation analyses were performed to evaluate whether UACR was involved in the relationship between urinary arsenic exposure and lipid metabolism outcomes. Associations involving the principal exposure indicators, UTA and DMA, were considered statistically significant with an FDR-adjusted *q* < 0.1 (34, 35). For exploratory analyses, a nominal *P* < 0.05 was considered indicative of a potential association (36). Before fitting the linear regression models, the normality of the residuals and homoscedasticity were assessed. Missing data in NHANES were handled using complete-case analysis. In the Zhaodong population, missing values for selected continuous covariates were imputed using the corresponding mean values, whereas participants with missing key exposure or outcome data were excluded from the relevant analyses. Statistical analyses were conducted with R software (version 4.4.2; R Foundation for Statistical Computing, Vienna, Austria).

## 3 Results

### 3.1 Baseline characteristics and urinary arsenic exposure levels

**Table 1** presents the baseline characteristics of the weighted NHANES population and the Zhaodong population. The NHANES analysis included 2,104 participants, representing a weighted population of 52,612,536 adults, while the Zhaodong analysis included 310 participants. Compared with the NHANES population, participants in the Zhaodong population were older, had a higher proportion of women, lower BMI, higher levels of UACR, TG, and non-HDL-C, and lower LDL-C levels.

**Table 1:** Baseline characteristics of participants in NHANES and the Zhaodong population.

Characteristics	NHANES, weighted (n =2104) (N = 52612536)	Zhaodong population (n =310)
Age, n (%)		
20-39	495 (26.67)	10 (3.23)
40-60	766 (41.81)	129 (41.61)
> 60	843 (31.52)	171 (55.16)
Sex, n (%)		
Female	857 (37.61)	190 (61.29)
Male	1247 (62.39)	120 (38.71)
BMI (kg/m^2^)	29.20 (24.73-33.70)	24.80 (23.00-26.80)
Race ^a^/Nationality ^b^, n (%)		
Mexican American ^a^	271 (5.84)	–
Other Hispanic ^a^	197 (3.96)	–
Non-Hispanic White ^a^	1106 (77.63)	–
Non-Hispanic Black ^a^	359 (7.46)	–
Other Race - Including Multi-Racial ^a^	171 (5.11)	–
Han Chinese ^b^	–	292 (94.19)
Other ethnic minorities ^b^	–	18 (5.81)
Education, n (%)		
Less Than 9th Grade	205 (5.04)	207 (66.77)
9-11th Grade (Includes 12th grade with no diploma)	289 (9.33)	79 (25.48)
High School Grad/GED or Equivalent	501 (24.58)	24 (7.74)
Some College or AA degree	686 (33.96)	
College Graduate or above	423 (27.09)	
PIR ^a/^Income ^b^, n (%)		
≤1.3 ^a^/< 20000 ^b^	716 (22.11)	206 (66.45)
1.3-3.5 ^a^/20000–60000 ^b^	794 (37.43)	86 (27.74)
>3.5 ^a^/> 60000 ^b^	594 (40.47)	18 (5.81)
Cotinine ^a^/Smoking ^b^, n (%)		
< 0.05 ^a^/Never ^b^	1136 (57.00)	211 (68.06)
0.05–10 ^a^/Ever ^b^	410 (16.53)	17 (5.48)
> 10 ^a^/Now ^b^	558 (26.48)	82 (26.45)
Hypertension, n (%)		
No	939 (51.62)	190 (61.29)
Yes	1165 (48.38)	120 (38.71)
Diabetes, n (%)		
No	1558 (79.36)	-
Yes	546 (20.64)	-
eGFR (mL/min/1.73 m²)	94.34 (79.16-106.99)	101.77 (93.24-112.03)
Seafood intake frequency, n (%)		
No	-	171 (55.16)
Once a day	-	1 (0.32)
At least once a week	-	28 (9.03)
At least once a month	-	72 (23.23)
At least once a year	-	38 (12.26)
AsB (μg/L)	0.78 (0.57-0.99)	–
Cycles, n (%)		
2009-2010	442 (15.51)	-
2011-2012	344 (18.35)	-
2013-2014	384 (18.00)	-
2015-2016	374 (18.97)	-
2017-2020	560 (29.17)	-
UCr (mg/dL)	82.00 (45.00-144.00)	23.81 (16.16-30.03)
UACR (mg/g)	7.20 (4.83-13.21)	45.24 (26.40-65.61)
TG (mg/dL)	105.00 (74.00-150.00)	145.25 (97.65-198.62)
LDL-C (mg/dL)	114.00 (90.00-140.00)	103.06 (87.01-120.94)
Non-HDL-C (mg/dL)	137.00 (112.85-165.97)	151.97 (129.25-174.98)
Urinary total arsenic (μg/L)	3.73 (2.23-6.08)	12.37 (7.32-18.81)
MMA (μg/L)	0.76 (0.49-1.13)	1.68 (0.99-2.53)
DMA (ug/L)	3.48 (2.53-4.98)	8.72 (5.13-15.37)
Arsenite (As^III^) (μg/L)	0.55 (0.38-0.80)	1.76 (1.25-2.53)
Arsenate (As^V^) (μg/L)	1.05 (0.92-1.27)	1.55 (1.17-2.77)

^a^Indicates variables derived from NHANES; ^b^Indicates variables derived from the Zhaodong population. For NHANES, n represents the unweighted sample size, whereas percentages and continuous variables were estimated using survey weights. For descriptive purposes, baseline medians and interquartile ranges for urinary arsenic variables were calculated based on measured values at or above the limit of detection, reflecting the actual concentration distribution among detectable samples.

UTA levels were higher in Zhaodong than in NHANES, with median concentrations of 12.37 μg/L and 3.73 μg/L, respectively. Similar differences were observed for individual species: MMA, 0.76 versus 1.68 μg/L; DMA, 3.48 versus 8.72 μg/L; As^III^, 0.55 versus 1.76 μg/L; and As^V^, 1.05 versus 1.55 μg/L. In NHANES, values below the LOD accounted for 71.6% of As^III^ measurements, 97.4% of As^V^, 63.7% of MMA, and 38.6% of DMA. In the Zhaodong population, the corresponding proportions were 19.4%, 95.5%, 82.2%, and 0%, respectively. After values below the LOD were replaced with LOD/√2 and all observations were included, the corresponding median concentrations in NHANES and Zhaodong were 0.59 and 0.35 μg/L for MMA, 2.24 and 8.72 μg/L for DMA, 0.34 and 1.53 μg/L for As^III^, and 0.56 and 0.57 μg/L for As^V^, respectively.

#### 3.1.1 Baseline characteristics across urinary arsenic quartiles

Weighted baseline characteristics of the NHANES sample according to quartiles of creatinine-adjusted UTA and DMA are shown in **Table 2**. Sex, race/ethnicity, BMI, urinary creatinine, survey cycle, education level, tobacco exposure, and eGFR differed across quartiles of UTA or DMA, although the patterns of these differences were not entirely consistent between the two urinary arsenic indicators. Detailed results are provided in **Supplementary Tables 2, 3**.

**Table 2:** Summary of P values for baseline characteristics across quartiles of creatinine-corrected urinary total arsenic and arsenic species in NHANES and the Zhaodong population.

Characteristics	NHANES, weighted		Zhaodong population	
	UTA	DMA	UTA	DMA
Sex, (*P*)	<0.001	<0.001	0.082	0.022
Age, (*P*)	0.050	0.001	0.509	0.405
Race ^a^, (*P*) Nationality ^b^, (*P*)	<0.001	<0.001	0.382	0.382
Education, (*P*)	0.011	0.023	0.841	0.782
PIR ^a^, (*P*) Income ^b^, (*P*)	0.133	0.126	0.342	0.070
Cotinine ^a^, (*P*) Smoking ^b^, (*P*)	0.003	<0.001	0.048	0.032
Hypertension, (*P*)	0.624	0.540	0.319	0.691
Diabetes, (*P*)	0.384	0.558	–	–
BMI, (*P*)	<0.001	<0.001	0.806	0.721
EGFR, (*P*)	0.003	0.322	0.046	0.207
Urinary AsB ^a^, (*P*) Seafood ^b^, (*P*)	0.084	0.364	0.048	0.655
Cycles, (*P*)	<0.001	0.277	–	–

^a^Indicates variables derived from NHANES; ^b^Indicates variables derived from the Zhaodong population. Quartiles were defined separately according to creatinine-corrected urinary total arsenic and each arsenic species within each population. Creatinine-corrected urinary arsenic concentrations were calculated as urinary arsenic concentration (μg/L) × 100/urinary creatinine (mg/dL), and expressed as μg/g creatinine. UTA, urinary total arsenic; DMA, dimethylarsinic acid.

**Table 2** also shows that, compared with the NHANES sample, fewer baseline characteristics differed across quartiles of UTA and DMA in the Zhaodong population. Smoking status differed across quartiles of both UTA and DMA, whereas eGFR and the frequency of aquatic product or seafood consumption differed only across quartiles of UTA. Detailed results are shown in **Supplementary Tables 4 and 5**.

### 3.2 Associations of urinary arsenic exposure with lipid metabolism indicators

#### 3.2.1 NHANES population

**Table 3** summarizes the weighted linear regression analyses of creatinine-adjusted UTA and DMA in relation to lipid metabolism indicators in NHANES. In crude models, no consistent linear associations were observed for TG, LDL-C, or non-HDL-C. After covariate adjustment, UTA and DMA were positively associated with LDL-C. Each 1 μg/g creatinine increase in UTA and DMA corresponded to higher LDL-C levels of 0.68 mg/dL (95% CI: 0.09, 1.26; *P* = 0.027; *q* < 0.1) and 0.85 mg/dL (95% CI: 0.09, 1.61; *P* = 0.033; *q* < 0.1), respectively. Neither UTA nor DMA was significantly associated with TG or non-HDL-C after multivariable adjustment, and trend tests were not significant.

**Table 3:** Crude and adjusted linear associations between creatinine-corrected urinary total arsenic and arsenic species and lipid metabolism indicators in NHANES and the Zhaodong population.

Characteristics	Model	NHANES, weighted			Zhaodong population		
		β (95%CI)	*P (q)*	*P(q)- for trend*	β (95%CI)	*P (q)*	*P(q)- for trend*
TG							
UTA	Model 1	-0.11 (-1.20 ~ 0.97)	0.837 (0.885)	0.565 (0.678)	0.18 (-0.01 ~ 0.37)	0.063 (0.261)	0.359 (0.742)
	Model 2	0.63 (-0.31 ~ 1.57)	0.193 (0.234)	0.455 (0.796)	0.18 (-0.01 ~ 0.37)	0.068 (0.348)	0.309 (0.500)
DMA	Model 1	-0.11 (-1.56 ~ 1.34)	0.885 (0.885)	0.505 (0.678)	0.25 (-0.04 ~ 0.54)	0.087 (0.261)	0.448 (0.742)
	Model 2	0.87 (-0.43 ~ 2.17)	0.195 (0.234)	0.711 (0.796)	0.24 (-0.06 ~ 0.53)	0.116 (0.348)	0.577 (0.577)
LDL-C							
UTA	Model 1	0.52 (-0.15 ~ 1.19)	0.131 (0.393)	0.681 (0.681)	-0.01 (-0.06 ~ 0.04)	0.669 (0.669)	0.495 (0.742)
	Model 2	0.68 (0.09 ~ 1.26)	0.027 (0.099)	0.360 (0.796)	-0.02 (-0.08 ~ 0.03)	0.399 (0.599)	0.184 (0.500)
DMA	Model 1	0.78 (-0.12 ~ 1.67)	0.092 (0.393)	0.418 (0.678)	-0.02 (-0.10 ~ 0.06)	0.615 (0.669)	0.414 (0.742)
	Model 2	0.85 (0.09 ~ 1.61)	0.033 (0.099)	0.271 (0.796)	-0.04 (-0.12 ~ 0.04)	0.338 (0.599)	0.146 (0.500)
Non-HDL-C							
UTA	Model 1	0.11 (-0.46 ~ 0.67)	0.710 (0.885)	0.512 (0.678)	0.02 (-0.05 ~ 0.08)	0.624 (0.669)	0.834 (0.913)
	Model 2	0.43 (-0.05 ~ 0.91)	0.082 (0.164)	0.796 (0.796)	0.01 (-0.07 ~ 0.07)	0.986 (0.987)	0.333 (0.500)
DMA	Model 1	-0.23 (-0.97 ~ 0.51)	0.544 (0.885)	0.164 (0.678)	0.03 (-0.07 ~ 0.13)	0.597 (0.669)	0.913 (0.913)
	Model 2	0.20 (-0.45 ~ 0.85)	0.543 (0.543)	0.641 (0.796)	-0.01 (-0.11 ~ 0.10)	0.987 (0.987)	0.514 (0.577)

NHANES: Model 1: Unadjusted (Crude); Model 2: Adjusted (Sex, Age, Race, Education, PIR, Cotinine, Hypertension, BMI, eGFR, Urinary AsB, survey cycle). Zhaodong population: Model 1: Unadjusted (Crude); Model 2: Adjusted (Age, Sex, Ethnicity, Education, Income, BMI, eGFR, smoking status, frequency of aquatic product or seafood consumption, Hypertension). UTA, urinary total arsenic; DMA, dimethylarsinic acid. q, Benjamini–Hochberg false discovery rate-adjusted P value; q for trend, Benjamini–Hochberg false discovery rate-adjusted P value for trend.

**Supplementary Table 6** presents the logistic regression results for lipid abnormalities. After full adjustment, no significant associations were found between quartiles of UTA or DMA and abnormal TG, LDL-C, or non-HDL-C in NHANES.

#### 3.2.2 Zhaodong population

In the Zhaodong population, neither UTA nor DMA was significantly associated with TG, LDL-C, or non-HDL-C in the adjusted linear regression models, and trend tests were non-significant (**Table 3**).

As shown in **Supplementary Table 6**, the highest quartile of DMA was associated with lower odds of abnormal LDL-C compared with Q1 (OR = 0.28, 95% CI: 0.10–0.79; *P* = 0.016; *q* < 0.1). The highest quartile of UTA also showed lower odds of abnormal LDL-C (OR = 0.33, 95% CI: 0.12–0.93; *P* = 0.037; *q* ≈ 0.1), although the association was borderline after FDR correction. No significant associations were observed for abnormal TG or non-HDL-C.

### 3.3 Dose–response relationships of urinary arsenic exposure with lipid metabolism indicators

#### 3.3.1 NHANES population

**Figure 1:**
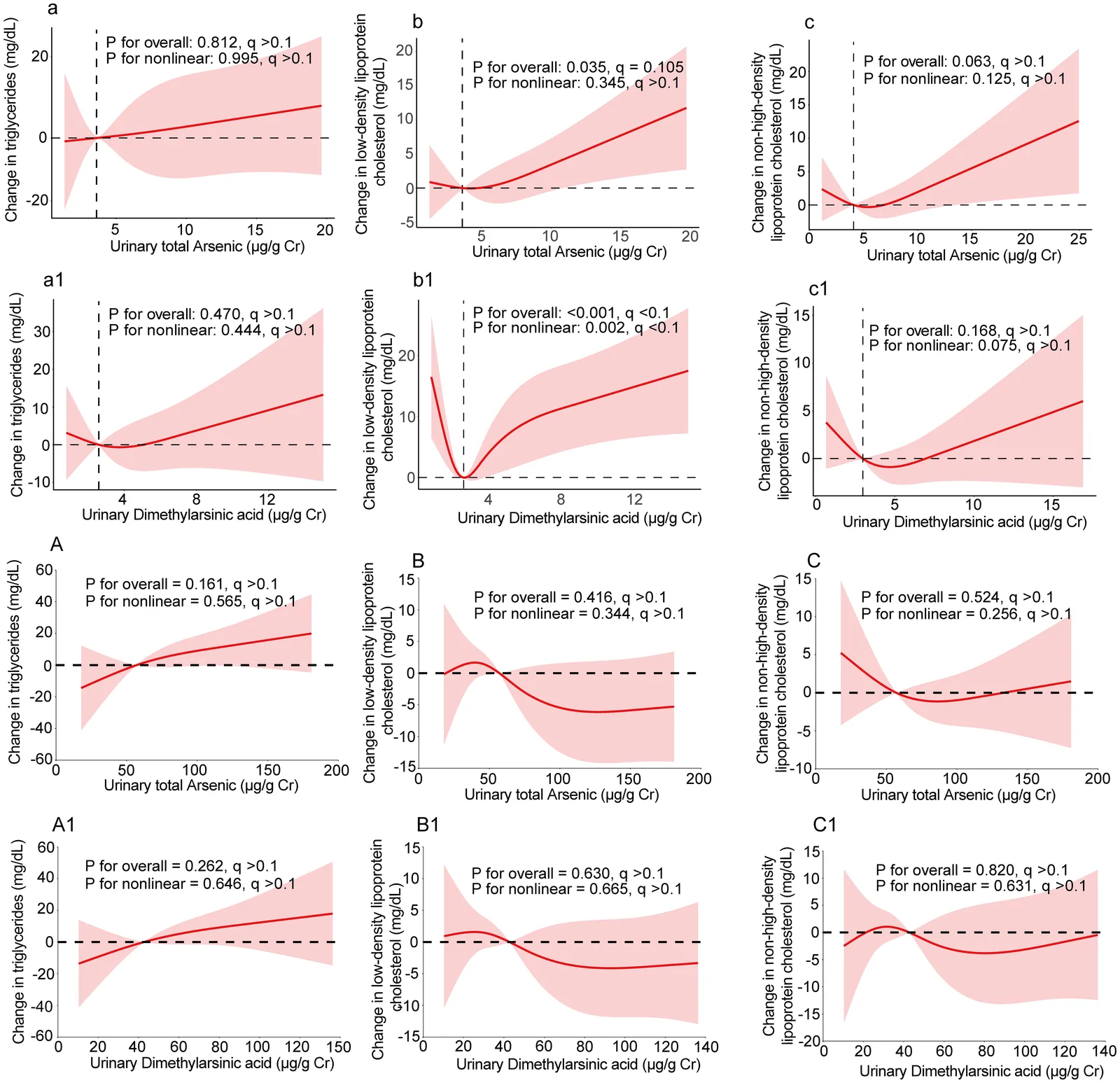
Restricted cubic spline analyses of the associations of urinary total arsenic and dimethylarsinic acid with TG, LDL-C, and non-HDL-C in survey-weighted NHANES and the Zhaodong population. Panels **(a–c, a1–c1)** represent UTA and DMA in NHANES, respectively; panels **(A–C, A1–C1)** represent UTA and DMA in Zhaodong, respectively. Models were adjusted for demographic, socioeconomic, lifestyle, renal, and seafood-related covariates, as applicable, as detailed in the Methods. Sample sizes were 959, 950, and 2,104 for TG, LDL-C, and non-HDL-C in NHANES, respectively, and 310 in Zhaodong. Solid lines represent the estimated associations, and shaded areas represent 95% confidence intervals.

RCS-derived exposure–response curves for UTA and DMA are shown in **Figure 1**. In NHANES, LDL-C showed exposure–response signals for both UTA and DMA. UTA was associated with LDL-C overall (*P* for overall = 0.035; *q* ≈ 0.1), without evidence of nonlinearity (*P* for nonlinear = 0.345). The curve suggested that LDL-C began to increase when UTA exceeded 3.72 μg/g Cr. For DMA, both the overall and nonlinear associations with LDL-C remained significant after FDR correction (*P* for overall <0.001; *q* < 0.1; *P* for nonlinear = 0.002; *q* < 0.1), with LDL-C levels increasing gradually above 2.59 μg/g Cr. Neither UTA nor DMA showed significant exposure–response patterns with TG or non-HDL-C.

#### 3.3.2 Zhaodong population

In the Zhaodong population, neither the overall associations nor the tests for nonlinearity between UTA or DMA and TG, LDL-C, or non-HDL-C reached statistical significance. Detailed results are shown in **Figure 1**.

### 3.4 Associations among urinary arsenic exposure, UACR, and lipid metabolism indicators

#### 3.4.1 NHANES population

In NHANES, multivariable-adjusted models showed positive associations of UTA and DMA with UACR, and both associations remained statistically significant after FDR correction (*q* < 0.1). Significant trends were also observed for UTA and DMA (*q* for trend < 0.1). We further examined the relationships between UACR and lipid metabolism indicators. UACR was inversely associated with LDL-C after covariate adjustment (β = −0.01; *P* = 0.002; *q* < 0.1). UACR was not significantly associated with TG in the continuous linear regression analysis, whereas the trend test was significant (*P* for trend = 0.021; *q* for trend < 0.1). No significant association was observed between UACR and non-HDL-C. Detailed results are presented in **Table 4**.

**Table 4:** Crude and adjusted linear associations of creatinine-corrected urinary total arsenic and arsenic species with UACR, and associations of UACR with lipid metabolism indicators in NHANES and the Zhaodong population.

Characteristics	Model	NHANES, weighted			Zhaodong population		
		β (95%CI)	*P (q)*	*P (q)- for trend*	β (95%CI)	*P (q)*	*P (q)- for trend*
UTA to UACR							
	Model 1	0.14 (-1.37 ~ 1.65)	0.854 (0.854)	0.747 (0.783)	0.01 (-0.08 ~ 0.09)	0.870 (0.870)	0.461 (0.461)
	Model 2	2.39 (0.42 ~ 4.35)	0.020 (0.022)	0.032 (0.064)	-0.04 (-0.12 ~ 0.05)	0.396 (0.610)	0.999 (0.999)
DMA to UACR							
	Model 1	0.24 (-1.89 ~ 2.37)	0.825 (0.854)	0.783 (0.783)	0.03 (-0.10 ~ 0.16)	0.635 (0.870)	0.434 (0.461)
	Model 2	2.97 (0.49 ~ 5.46)	0.022 (0.022)	0.093 (0.093)	-0.03 (-0.16 ~ 0.10)	0.610 (0.610)	0.917 (0.999)
UACR to TG							
	Model 1	0.08 (-0.02 ~ 0.18)	0.135 (0.203)	0.025 (0.075)	0.49 (0.25 ~ 0.73)	<0.001 (<0.003)	0.001 (0.003)
	Model 2	0.01 (-0.08 ~ 0.11)	0.770 (0.770)	0.021 (0.063)	0.45 (0.19 ~ 0.70)	<0.001 (<0.003)	0.008 (0.024)
UACR to LDL-C							
	Model 1	-0.01 (-0.99 ~ -0.01)	<0.001 (<0.003)	0.057 (0.086)	0.04 (-0.03 ~ 0.11)	0.268 (0.268)	0.522 (0.522)
	Model 2	-0.01 (-0.01 ~ -0.01)	0.002 (0.006)	0.070 (0.105)	0.01 (-0.06 ~ 0.09)	0.780 (0.780)	0.958 (0.958)
UACR to Non-HDL-C							
	Model 1	0.01 (-0.01 ~ 0.02)	0.358 (0.358)	0.634 (0.634)	0.11 (0.02 ~ 0.19)	0.016 (0.024)	0.151 (0.226)
	Model 2	0.00 (-0.01 ~ 0.02)	0.573 (0.770)	0.650 (0.650)	0.06 (-0.03 ~ 0.15)	0.207 (0.310)	0.603 (0.904)

NHANES: Model 1: Unadjusted (Crude); Model 2: Adjusted (Sex, Age, Race, Education, PIR, Cotinine, Hypertension, BMI, eGFR, Urinary AsB, survey cycle). Zhaodong population: Model 1: Unadjusted (Crude); Model 2: Adjusted (Age, Sex, Nationality, ethnicity, Income, BMI, eGFR, smoking status, frequency of aquatic product or seafood consumption, Hypertension). UTA, urinary total arsenic; DMA, dimethylarsinic acid.q, Benjamini–Hochberg false discovery rate-adjusted P value; q for trend, Benjamini–Hochberg false discovery rate-adjusted P value for trend.

#### 3.4.2 Zhaodong population

In the Zhaodong population, neither UTA nor DMA showed significant adjusted linear associations with UACR (**Table 4**). UACR was positively associated with TG after covariate adjustment (β = 0.45; *P* < 0.001; *q* < 0.1), and the corresponding trend test was also significant (*P* for trend = 0.008; *q* for trend < 0.1). No significant adjusted associations were observed between UACR and LDL-C or non-HDL-C.

### 3.5 Mediation analyses involving UACR

#### 3.5.1 Mediation analysis in the NHANES sample

**Table 5** summarizes the mediation analyses involving UACR. In NHANES, significant negative indirect effects through UACR were observed only in the LDL-C models involving UTA and DMA, with ACMEs of −0.02 and −0.04, respectively (both *P* < 0.05; both *q* < 0.1), corresponding to mediated proportions of −3.28% and −3.83%. Direct and total effects were also significant for UTA and DMA in the LDL-C models (all *P* < 0.05; all *q* < 0.1). No significant indirect effects were observed for the other exposure–outcome combinations.

**Table 5:** Mediation analysis of UACR in the associations between urinary total arsenic, arsenic species, and lipid metabolism indicators in the NHANES and Zhaodong populations.

Exposure	Mediator	Outcome	ACME 95% CI	*P (q)*	ADE 95% CI	*P (q)*	Total effect 95% CI	*P (q)*	Proportion Of mediation (%)
NHANES, weighted									
UTA	UACR	TG	0.06 (-0.06 ~ 0.24)	0.402 (0.584)	0.58 (-0.36 ~ 1.48)	0.228 (0.274)	0.64 (-0.31 ~ 1.56)	0.194 (0.233)	5.74
		LDL-C	-0.02 (-0.06 ~ -0.00)	0.033 (0.099)	0.70 (0.20 ~ 1.27)	0.020 (0.060)	0.67 (0.16 ~ 1.24)	0.020 (0.060)	-3.28
		Non-HDL-C	0.01 (-0.02 ~ 0.05)	0.584 (0.584)	0.41 (-0.06 ~ 0.91)	0.088 (0.176)	0.42 (-0.05 ~ 0.91)	0.086 (0.172)	1.57
DMA	UACR	TG	0.08 (-0.09 ~ 0.33)	0.378 (0.584)	0.86 (-0.48 ~ 2.24)	0.218 (0.274)	0.94 (-0.43 ~ 2.33)	0.188 (0.233)	6.62
		LDL-C	-0.04 (-0.08 ~ -0.00)	0.030 (0.099)	0.90 (0.17 ~ 1.67)	0.012 (0.060)	0.87 (0.14 ~ 1.64)	0.016 (0.060)	-3.83
		Non-HDL-C	0.01 (-0.03 ~ 0.06)	0.576 (0.584)	0.18 (-0.46 ~ 0.86)	0.582 (0.582)	0.19 (-0.45 ~ 0.86)	0.554 (0.554)	1.50
Zhaodong population									
UTA	UACR	TG	-0.02 (-0.07 ~ 0.02)	0.416 (0.954)	0.19 (0.01 ~ 0.38)	0.040 (0.240)	0.18 (-0.01 ~ 0.37)	0.060 (0.300)	-7.43
		LDL-C	-0.0003 (-0.005 ~ 0.004)	0.902 (0.954)	-0.0234 (-0.076 ~ 0.031)	0.370 (0.555)	-0.0237 (-0.076 ~ 0.031)	0.364 (0.546)	-0.161
		Non-HDL-C	-0.0021 (-0.011 ~ 0.004)	0.546 (0.954)	0.0024 (-0.063 ~ 0.070)	0.956 (0.992)	0.0002 (-0.065 ~ 0.069)	0.996 (0.996)	0.280
DMA	UACR	TG	-0.015 (-0.09 ~ 0.04)	0.618 (0.954)	0.25 (-0.03 ~ 0.53)	0.082 (0.246)	0.24 (-0.06 ~ 0.52)	0.100 (0.300)	-4.10
		LDL-C	-0.0003 (-0.007 ~ 0.005)	0.954 (0.954)	-0.0407 (-0.122 ~ 0.041)	0.328 (0.555)	-0.0410 (-0.123 ~ 0.040)	0.328 (0.546)	0.030
		Non-HDL-C	-0.0019 (-0.015 ~ 0.007)	0.712 (0.954)	0.0009 (-0.100 ~ 0.102)	0.992 (0.992)	-0.0010 (-0.105 ~ 0.100)	0.972 (0.996)	0.367

ACME, average causal mediation effect; ADE, average direct effect; CI, confidence interval; UACR, urinary albumin-to-creatinine ratio; UTA, urinary total arsenic; DMA, dimethylarsinic acid; TG, triglycerides; LDL-C, low-density lipoprotein cholesterol; non-HDL-C, non-high-density lipoprotein cholesterol; q, Benjamini–Hochberg false discovery rate-adjusted P value.

#### 3.5.2 Mediation analysis in the Zhaodong population

In the Zhaodong population, no significant UACR-mediated indirect effects were observed in the TG, LDL-C, or non-HDL-C models involving UTA or DMA. Detailed estimates are provided in **Table 5**.

### 3.6 Supplementary exploratory analyses

In NHANES, RCS analyses suggested nominal exposure–response associations of As^V^ with LDL-C and of As^III^ with non-HDL-C. MMA, As^III^, and As^V^ were positively associated with UACR, and a nominal negative indirect effect through UACR was observed in the MMA–LDL-C model. In the Zhaodong population, As^V^ showed a nominal positive linear association with TG, whereas higher As^III^ quartiles were associated with greater odds of abnormal non-HDL-C. Mediation analyses also indicated nominal direct and total effects of As^V^ on TG. Detailed results are provided in **Supplementary Tables 7–17** and **Supplementary Figure 3**.

### 3.7 Subgroup analyses

**Supplementary Figure 4** presents subgroup analyses stratified by age, sex, BMI, hypertension status, and smoking-related exposure. In NHANES, a nominal interaction by hypertension status was observed for the association between UTA and LDL-C, with a positive association mainly among participants with hypertension. Associations of MMA, As^III^, and As^V^ with selected lipid metabolism indicators also showed nominal interactions by sex or BMI, although most stratum-specific estimates were not statistically significant. In the Zhaodong population, a nominal interaction by age was observed for the association between DMA and TG, although the associations within individual age strata were not statistically significant. The association between As^V^ and non-HDL-C showed a nominal interaction by hypertension status, with a positive association among participants with hypertension. No consistent evidence of effect modification was observed in the remaining subgroup analyses.

## 4 Discussion

In this cross-sectional study using data from NHANES and an independent adult population from Zhaodong, China, we examined the associations of urinary arsenic biomarkers with lipid metabolism indicators and UACR under low-to-moderate arsenic exposure conditions. We also explored whether UACR was involved in the relationships between urinary arsenic exposure and lipid metabolism. In NHANES, the principal exposure indicators, UTA and DMA, were positively associated with LDL-C, and DMA showed a nonlinear exposure–response relationship with LDL-C. UTA and DMA were also positively associated with UACR. However, the positive associations of UTA and DMA with LDL-C were not replicated in the Zhaodong population. In the adjusted linear models, neither UTA nor DMA was significantly associated with LDL-C, whereas the logistic regression analyses showed lower odds of abnormal LDL-C in the highest exposure quartiles. Taken together, these findings indicate heterogeneity in the associations of UTA and DMA with LDL-C across populations and analytical approaches, which may reflect differences in population context and outcome definition. Some secondary findings were broadly similar between the two populations, including the absence of significant associations of UTA and DMA with TG or non-HDL-C and the lack of a meaningful mediating role of UACR. Supplementary exploratory analyses identified several nominal species-specific associations, including As^V^ with TG and As^III^ with abnormal non-HDL-C in Zhaodong. However, both the As^V^–TG and MMA–TG estimates were imprecise, as indicated by their wide confidence intervals, and should therefore be interpreted cautiously. The As^V^–TG estimate was further limited by the high proportion of As^V^ measurements below the LOD. Overall, these cross-sectional findings suggest exploratory associations between urinary arsenic biomarkers and lipid metabolism indicators, particularly the associations of UTA and DMA with LDL-C observed in NHANES, while also indicating potential population- and species-specific heterogeneity that warrants confirmation in larger, independent studies, including prospective studies.

Among the lipid metabolism indicators examined, LDL-C showed the clearest arsenic-related signals, particularly in NHANES. Previous NHANES analyses have linked urinary arsenic indicators, including UTA and selected species, to cholesterol-related outcomes such as TC and LDL-C (14). This is consistent with our NHANES finding that UTA and DMA were related to higher LDL-C levels. LDL-C plays a central role in atherogenesis, as higher LDL-C concentrations facilitate cholesterol accumulation within the arterial wall, endothelial dysfunction, and plaque development (8, 9). Previous systematic reviews and dose–response analyses have linked chronic arsenic exposure to higher risks of coronary heart disease, stroke, and other cardiovascular outcomes, although estimates vary by exposure level, exposure source, and outcome definition (14, 15). Compared with evidence on cardiovascular outcomes, evidence regarding earlier lipid metabolic changes under low-to-moderate arsenic exposure conditions remains even more limited, particularly for individual urinary arsenic species and lipid indicators such as non-HDL-C and TG. Therefore, the LDL-C-related findings observed in NHANES may reflect a potential epidemiological association between arsenic exposure and atherosclerosis-related lipid profiles. The different association patterns observed in the Zhaodong population further suggest that this relationship may vary according to population context, exposure characteristics, and outcome definition and requires confirmation in larger independent populations.

Different urinary arsenic species may differ in exposure sources, metabolic pathways, and toxicological profiles. The DMA–LDL-C relationship was the clearest arsenic species-specific signal in this study, with the RCS analysis indicating a nonlinear exposure–response pattern. DMA, the predominant methylated arsenic species in urine, is often regarded as an indicator of arsenic methylation capacity and may also reflect biological responses to arsenic exposure (2, 3). The spline curve suggested that estimated LDL-C levels were lower across the lower range of DMA concentrations and gradually increased at higher DMA concentrations, indicating a possible nonlinear association rather than a strictly linear pattern. Beyond DMA, supplementary RCS analyses indicated nonlinear patterns for As^V^ with LDL-C and for As^III^ with non-HDL-C, suggesting species-specific relationships with lipid metabolism indicators. However, As^III^, As^V^, and MMA had relatively high proportions of values below the LOD in some samples, particularly As^V^. Therefore, these findings should be interpreted with caution and confirmed in larger studies with more reliable measurements of urinary arsenic species.

Interpretation of the findings for urinary arsenic species should also consider differences in arsenic sources and metabolism. UTA reflects the overall urinary arsenic burden, but it may include inorganic arsenic, methylated arsenic metabolites, and arsenic from some organic sources. Previous NHANES studies have shown that seafood consumption can substantially influence UTA, DMA, and AsB levels; therefore, the potential contribution of seafood-derived organic arsenic should be carefully controlled when interpreting UTA and DMA in general population studies (29, 37, 38). In this study, NHANES models adjusted for urinary AsB, whereas the Zhaodong analyses accounted for frequency of aquatic product or seafood consumption. Nevertheless, residual influence from dietary organic arsenic and its metabolites on urinary arsenic species cannot be excluded. Therefore, DMA may reflect inorganic arsenic methylation, but it may also be partly influenced by dietary organic arsenic, increasing uncertainty in the interpretation of DMA-related findings across studies and populations.

Notably, the urinary arsenic–lipid metabolism associations in the Zhaodong population only partly aligned with those observed in NHANES. Linear regression showed positive associations of UTA and DMA with LDL-C in NHANES, but these patterns were not evident in the Zhaodong population. In contrast, logistic regression models indicated lower odds of abnormal LDL-C among participants in the highest quartiles of UTA and DMA in the Zhaodong population. This inverse association was not consistently supported by the linear regression or RCS analyses and was not replicated in the NHANES sample. This discrepancy may partly reflect the limited sample size of the Zhaodong population, differences in participant characteristics, and the possibility of residual confounding. The two populations also differed in demographic composition, including age, sex distribution, BMI, and baseline lipid profiles. In addition, different laboratory platforms were used for urinary arsenic measurements, with ICP-MS used in NHANES and HG-AFS or HPLC-HG-AFS used in Zhaodong, which may have affected analytical sensitivity and cross-population comparability. In particular, under low-to-moderate arsenic exposure conditions, DMA may be influenced by both inorganic arsenic methylation capacity and dietary organic arsenic sources (37, 38). Unlike the NHANES analysis, in which participants with relatively high urinary AsB levels were excluded and urinary AsB was further adjusted for, the Zhaodong analysis mainly controlled for dietary organic arsenic exposure by adjusting for the frequency of aquatic product or seafood consumption. Thus, residual influence from seafood-derived organic arsenic may still exist. Differences in other exposure sources, dietary patterns, medication use, occupational exposure, and unmeasured population characteristics may also have contributed to the inconsistent findings. Meanwhile, supplementary exploratory analyses showed nominal associations of As^V^ with higher TG and of elevated As^III^ quartiles with greater odds of abnormal non-HDL-C in the Zhaodong population. However, the As^V^–TG estimate was imprecise, as indicated by its wide confidence interval, and should be interpreted cautiously given the high proportion of As^V^ measurements below the LOD. Overall, the findings from the Zhaodong population are more appropriately interpreted as exploratory and may reflect population- and species-specific heterogeneity.

A further finding from NHANES was the positive association of the principal exposure indicators, UTA and DMA, with UACR. Supplementary exploratory analyses also suggested nominal positive associations of MMA, As^III^, and As^V^ with UACR. As a marker of albuminuria and early glomerular injury, UACR has also been associated with systemic microvascular damage and endothelial dysfunction (39, 40). Previous population-based studies have reported associations of higher urinary arsenic levels with albuminuria, elevated UACR, and other markers of kidney injury (22–24). In addition, signals linking UACR to TG were observed in both study populations. In the NHANES sample, the adjusted linear association between UACR and TG did not reach statistical significance, although the trend test was significant. In the Zhaodong population, UACR was positively associated with TG levels. Previous studies have linked UACR more consistently with TG and TG-related lipid measures than with some cholesterol indicators, suggesting that TG may better capture metabolic disturbances related to increased urinary albumin excretion (18–20). Therefore, the observed UACR–TG associations may be consistent with shared biological processes related to renal microvascular injury and metabolic dysfunction, although the underlying mechanisms cannot be established from the present cross-sectional data.

The mediation analysis did not support a meaningful mediating role of UACR in the associations between urinary arsenic biomarkers and lipid metabolism indicators. In NHANES, small negative indirect effects through UACR were observed in the LDL-C models involving the principal exposure indicators UTA and DMA. A nominal negative indirect effect was also observed for MMA in the supplementary exploratory analysis. Given their small magnitudes and negative mediated proportions, these indirect effects were not considered to indicate a meaningful mediating role of UACR. The negative indirect effects arose because UTA and DMA were positively associated with UACR, whereas UACR was inversely associated with LDL-C. Consequently, the indirect pathway operated in the opposite direction to the positive direct and total effects of UTA and DMA on LDL-C. This pattern is therefore more consistent with statistical suppression or inconsistent mediation than with a classical mediation pathway. Importantly, these findings should not be interpreted as indicating a protective role of UACR or a biologically meaningful pathway linking arsenic exposure to LDL-C. Instead, they may reflect complex relationships among arsenic exposure, renal or microvascular function, and lipid metabolism, as well as residual confounding or reverse causation. Moreover, the cross-sectional design precludes determination of the temporal ordering among urinary arsenic exposure, UACR, and lipid metabolism indicators. Accordingly, these mediation findings should be interpreted as exploratory and require confirmation in future longitudinal studies (41).

Subgroup analyses examined whether age, sex, BMI, hypertension status, and tobacco-related factors modified the urinary arsenic–lipid metabolism associations. Although sex and BMI have been associated with arsenic methylation profiles, evidence for their modifying effects on arsenic-related metabolic outcomes remains inconclusive (42, 43). In this study, associations of MMA, As^III^, and As^V^ with selected lipid metabolism indicators showed interactions by sex or BMI; however, most stratum-specific estimates did not reach statistical significance. In addition, hypertension status may indicate potential susceptibility to arsenic-related cardiometabolic alterations. In NHANES, the UTA–LDL-C relationship varied by hypertension status, with the positive association primarily observed among participants with hypertension. In the Zhaodong population, a nominal interaction by hypertension status was observed for the As^V^–non-HDL-C association. Because subgroup analyses are subject to reduced sample size and multiple comparisons, these findings should be interpreted cautiously (44).

This study has several limitations. First, because urinary arsenic exposure, UACR, and lipid metabolism indicators were assessed cross-sectionally, their temporal ordering could not be established, and causal inference is not possible. Second, urinary arsenic concentrations were assessed using a single spot urine sample, which may have been influenced by short-term exposure and within-person variability and may therefore not accurately represent long-term arsenic exposure. Third, several urinary arsenic species had high proportions of measurements below the LOD, particularly As^V^, as well as MMA and As^III^ in NHANES. Substitution of values below the LOD with LOD/√2 resulted in substantial clustering at the substituted value, potentially reducing discrimination between exposure levels and affecting the robustness of species-specific regression, restricted cubic spline, and subgroup analyses. Because only a very small proportion of As^V^ measurements were detectable, analyses restricted to detected samples or based on detection status would have had limited statistical power and might also have yielded unstable estimates. Therefore, analyses involving MMA, As^III^, and As^V^ were considered supplementary and exploratory, and the corresponding findings should be interpreted cautiously. Fourth, although NHANES models adjusted for urinary AsB and the Zhaodong analyses considered the frequency of aquatic product or seafood consumption, detailed dietary information was not fully available. Therefore, residual confounding from seafood-derived organic arsenic, other dietary arsenic sources, and overall dietary patterns cannot be excluded. Fifth, the relatively small sample size of the Zhaodong population may have reduced statistical power for subgroup and species-specific analyses, warranting confirmation in larger populations. Moreover, the positive associations of UTA and DMA with LDL-C observed in NHANES were not replicated in the Zhaodong population, which limits the generalizability of these findings and suggests possible population-specific heterogeneity. Sixth, although much of the apparent reduction in the NHANES sample was attributable to the predefined one-third urinary arsenic laboratory subsampling design, subsequent exclusions may still have affected the representativeness of the final analytic sample. Consistent with previous NHANES studies of arsenic exposure, urinary AsB concentrations were restricted to reduce interference from seafood-derived organic arsenicals associated with recent seafood intake. However, this restriction may also have excluded some participants with relatively high UTA and DMA concentrations, thereby narrowing the distributions of urinary arsenic exposure. Excluding participants using lipid-lowering medications may also have underrepresented individuals with more severe dyslipidemia and reduced the variability of lipid outcomes. Although these selection criteria were intended to improve the specificity of exposure assessment and reduce the influence of pharmacological treatment on lipid outcomes, they may collectively have weakened the observed associations and led to underestimation of the underlying associations. In addition, the complete-case analysis used in NHANES may have introduced selection bias if participants with complete data differed systematically from those with incomplete data. Mean imputation of selected continuous covariates in the Zhaodong population may also have reduced variability and underestimated the uncertainty of the corresponding estimates. Finally, although the models accounted for multiple demographic, lifestyle, and clinical factors, residual confounding from diet, medication use, occupational exposure, genetic background, and other unmeasured factors remains possible.

## 5 Conclusions

Our findings suggest that, under low-to-moderate arsenic exposure conditions, UTA and DMA were associated with higher LDL-C and UACR in NHANES, with DMA showing a nonlinear exposure–response association with LDL-C. However, similar positive associations with LDL-C were not observed in the Zhaodong population, suggesting that these relationships may vary across population settings. Supplementary species-specific findings should be interpreted cautiously because of the high proportions of measurements below the LOD and the limited precision of some estimates. Mediation analyses did not support a clear or meaningful mediating role of UACR in arsenic–lipid associations. These findings are hypothesis-generating and should not be interpreted as evidence of causal relationships. Larger prospective studies with repeated exposure measurements and improved assessment of urinary arsenic species are needed to confirm these findings.

## Data Availability

The raw data supporting the conclusions of this article will be made available by the authors, without undue reservation.

## References

[1] World Health Organization . Arsenic. Available online at: https://www.who.int/zh/news-room/fact-sheets/detail/arsenic (Accessed March 20, 2026).

[2] El-Ghiaty MA El-Kadi AOS . The duality of arsenic metabolism: Impact on human health. Annu Rev Pharmacol Toxicol. (2023) 63:341–58. doi: 10.1146/annurev-pharmtox-051921-020936 36100221

[3] Caldwell KL Jones RL Verdon CP Jarrett JM Caudill SP Osterloh JD . Levels of urinary total and speciated arsenic in the US population: National Health and Nutrition Examination Survey 2003–2004. J Exposure Sci Environ Epidemiol. (2009) 19:59–68. doi: 10.1038/jes.2008.32 18523458

[4] Loomis D Guha N Hall AL Straif K . Identifying occupational carcinogens: an update from the IARC Monographs. Occup Environ Med. (2018) 75:593–603. doi: 10.1136/oemed-2017-104944 29769352 PMC6204931

[5] Shaji E Santosh M Sarath KV Prakash P Deepchand V Divya BV . Arsenic contamination of groundwater: A global synopsis with focus on the Indian Peninsula. Geosci Front. (2021) 12:101079. doi: 10.1016/j.gsf.2020.08.015 38826717

[6] Wang Y Ma L Wang C Gao T Han Y Xu DX . Cardiovascular adverse effects and mechanistic insights of arsenic exposure: a review. Environ Chem Lett. (2024) 22:1437–72. doi: 10.1007/s10311-023-01677-0 30311153

[7] Balarastaghi S Rezaee R Hayes AW Yarmohammadi F Karimi G . Mechanisms of arsenic exposure-induced hypertension and atherosclerosis: an updated overview. Biol Trace Elem Res. (2023) 201:98–113. doi: 10.1007/s12011-022-03153-2 35167029

[8] Ference BA Ginsberg HN Graham I Ray KK Packard CJ Bruckert E . Low-density lipoproteins cause atherosclerotic cardiovascular disease. 1. Evidence from genetic, epidemiologic, and clinical studies. A consensus statement from the European Atherosclerosis Society Consensus Panel. Eur Heart J. (2017) 38:2459–72. doi: 10.1093/eurheartj/ehx144 28444290 PMC5837225

[9] Mach F Koskinas KC Roeters van Lennep JE Tokgözoğlu L Badimon L Baigent C . 2025 Focused update of the 2019 ESC/EAS Guidelines for the management of dyslipidaemias. Eur Heart J. (2025) 46:4359–78. doi: 10.1093/eurheartj/ehaf190 40878289

[10] Rajagopalan S Brook RD Salerno PRVO Bourges-Sevenier B Landrigan P Nieuwenhuijsen MJ . Air pollution exposure and cardiometabolic risk. Lancet Diabetes Endocrinol. (2024) 12:196–208. doi: 10.1016/S2213-8587(23)00361-3 38310921 PMC11264310

[11] Zhao Y Li M Tian X Xie J Liu P Ying X . Effects of arsenic exposure on lipid metabolism: a systematic review and meta-analysis. Toxicol Mech Methods. (2021) 31:188–96. doi: 10.1080/15376516.2020.1864537 33472496

[12] Kuo CC Su PH Sun CW Liu HJ Chang CL Wang SL . Early-life arsenic exposure promotes atherogenic lipid metabolism in adolescence: a 15-year birth cohort follow-up study in central Taiwan. Environ Int. (2018) 118:97–105. doi: 10.1016/j.envint.2018.05.033 29859944

[13] Mendez MA González-Horta C Sánchez-Ramírez B Ballinas-Casarrubias L Hernández Cerón R Viniegra Morales D . Chronic exposure to arsenic and markers of cardiometabolic risk: a cross-sectional study in Chihuahua, Mexico. Environ Health Perspect. (2016) 124:104–11. doi: 10.1289/ehp.1408742 26068977 PMC4710594

[14] Qu C Huang R . Linking the low-density lipoprotein-cholesterol (LDL) level to arsenic acid, dimethylarsinic, and monomethylarsonic: Results from a national population-based study from the NHANES, 2003-2020. Nutrients. (2022) 14:3993. doi: 10.3390/nu14193993 36235646 PMC9573665

[15] Gopang M Yazdi MD Moyer A Smith DM Meliker JR . Low-to-moderate arsenic exposure: a global systematic review of cardiovascular disease risks. Environ Health. (2025) 24:29. doi: 10.1186/s12940-025-01184-5 40346670 PMC12065288

[16] Fengjun D Junli T Zilong L Xiaorong C Jiyu Z Jianwei X . Joint association of urinary albumin-to-creatinine ratio within normal range and triglyceride-glucose index with incident cardiovascular disease: a prospective cohort study. Cardiovasc Diabetol. (2025) 24:451. doi: 10.1186/s12933-025-03009-8 41316179 PMC12664133

[17] Claudel SE Waikar SS Schmidt IM Vasan RS Verma A . The relationship between low levels of albuminuria and mortality among adults without major cardiovascular risk factors. Eur J Prev Cardiol. (2024) 31:2046–55. doi: 10.1093/eurjpc/zwae189 38825979 PMC11629963

[18] Hwang SW Lee T Uh Y Lee JY . Urinary albumin creatinine ratio is associated with lipid profile. Sci Rep. (2024) 14:14870. doi: 10.1038/s41598-024-65037-w 38937496 PMC11211387

[19] Wang YX Wang AP Ye YN Gao ZN Tang XL Yan L . Elevated triglycerides rather than other lipid parameters are associated with increased urinary albumin to creatinine ratio in the general population of China: a report from the REACTION study. Cardiovasc Diabetol. (2019) 18:57. doi: 10.1186/s12933-019-0863-8 31054570 PMC6500581

[20] Xue J Wang Y Li B Yu S Wang A Wang W . Triglycerides to high-density lipoprotein cholesterol ratio is superior to triglycerides and other lipid ratios as an indicator of increased urinary albumin-to-creatinine ratio in the general population of China: a cross-sectional study. Lipids Health Dis. (2021) 20:13. doi: 10.1186/s12944-021-01442-8 33588849 PMC7883433

[21] Agrawal S Zaritsky JJ Fornoni A Smoyer WE . Dyslipidaemia in nephrotic syndrome: mechanisms and treatment. Nat Rev Nephrol. (2018) 14:57–70. doi: 10.1038/nrneph.2017.155 29176657 PMC5770189

[22] Zheng LY Umans JG Tellez-Plaza M Yeh F Francesconi KA Goessler W . Urine arsenic and prevalent albuminuria: evidence from a population-based study. Am J Kidney Dis. (2013) 61:385–94. doi: 10.1053/j.ajkd.2012.09.011 23142528 PMC3578134

[23] Rahman HH Niemann D Munson-McGee SH . Association of albumin to creatinine ratio with urinary arsenic and metal exposure: evidence from NHANES 2015-2016. Int Urol Nephrol. (2022) 54:1343–53. doi: 10.1007/s11255-021-03018-y 34643861

[24] Hasan NT Xu X Han D Sansom G Roh T . Association between urinary arsenic levels and kidney damage in US adults: NHANES 2007-2018. J Trace Elem Med Biol. (2024) 86:127559. doi: 10.1016/j.jtemb.2024.127559 39522452

[25] Centers for Disease Control and Prevention National Center for Health Statistics . National Health and Nutrition Examination Survey: About NHANES. Available online at: https://www.cdc.gov/nchs/nhanes/about/index.html (Accessed May 17, 2026).

[26] Jones MR Tellez-Plaza M Vaidya D Grau M Francesconi KA Goessler W . Estimation of inorganic arsenic exposure in populations with frequent seafood intake: evidence from MESA and NHANES. Am J Epidemiol. (2016) 184:590–602. doi: 10.1093/aje/kww097 27702745 PMC5065621

[27] Moon KA Guallar E Umans JG Devereux RB Best LG Francesconi KA . Association between exposure to low to moderate arsenic levels and incident cardiovascular disease. A prospective cohort study. Ann Intern Med. (2013) 159:649–59. doi: 10.7326/0003-4819-159-10-201311190-00719 24061511 PMC4157936

[28] Hsieh CY Wang SL Fadrowski JJ Navas-Acien A Kuo CC . Urinary concentration correction methods for arsenic, cadmium, and mercury: a systematic review of practice-based evidence. Curr Environ Health Rep. (2019) 6:188–99. doi: 10.1007/s40572-019-00242-8 31372861

[29] Nigra AE Moon KA Jones MR Sanchez TR Navas-Acien A . Urinary arsenic and heart disease mortality in NHANES 2003-2014. Environ Res. (2021) 200:111387. doi: 10.1016/j.envres.2021.111387 34090890 PMC8403626

[30] Hardy ST Sakhuja S Jaeger BC Urbina EM Suglia SF Feig DI . Trends in blood pressure and hypertension among US children and adolescents, 1999-2018. JAMA Netw Open. (2021) 4:e213917. doi: 10.1001/jamanetworkopen.2021.3917 33792732 PMC8017470

[31] Qi X Fu J Liu J Wu X Zheng X Wang S . Association between secondhand smoke exposure and rheumatoid arthritis in US never-smoking adults: a cross-sectional study from NHANES. Sci Rep. (2024) 14:11061. doi: 10.1038/s41598-024-61950-2 38745032 PMC11094008

[32] Levey AS Stevens LA Schmid CH Zhang YL Castro AF Feldman HI . A new equation to estimate glomerular filtration rate. Ann Intern Med. (2009) 150:604–12. doi: 10.7326/0003-4819-150-9-200905050-00006 19414839 PMC2763564

[33] Centers for Disease Control and Prevention . Adult Bmi Categories. Available online at: https://www.cdc.gov/bmi/adult-calculator/bmi-categories.html (Accessed May 16, 2026).

[34] Rabotnick MH Haidari A Dolinoy DC Meijer JL Harris SM Burant CF . Early pregnancy serum PFAS are associated with alterations in the maternal lipidome. Environ Res. (2024) 263:120183. doi: 10.1016/j.envres.2024.120183 39426451 PMC11639123

[35] Puttabyatappa M Banker M Zeng L Goodrich JM Domino SE Dolinoy DC . Maternal exposure to environmental disruptors and sexually dimorphic changes in maternal and neonatal oxidative stress. J Clin Endocrinol Metab. (2020) 105:492–505. doi: 10.1210/clinem/dgz063 31613966 PMC7046018

[36] Ismaeel A Fletcher E Papoutsi E Thomas NT Bohannon WT Pipinos II . Multiomics analysis of skeletal muscle identifies dysregulation of hypoxia-induced genes in peripheral artery disease. J Am Heart Assoc. (2025) 14:e042350. doi: 10.1161/JAHA.125.042350 41025488 PMC12684623

[37] Davydiuk T Tao J Lu X Le XC . Effects of dietary intake of arsenosugars and other organic arsenic species on studies of arsenic methylation efficiency in humans. Environ Health (Wash). (2023) 1:236–48. doi: 10.1021/envhealth.3c00090 37881591 PMC10594586

[38] Taylor VF Li Z Sayarath V Palys TJ Morse KR Scholz-Bright RA . Distinct arsenic metabolites following seaweed consumption in humans. Sci Rep. (2017) 7:3920. doi: 10.1038/s41598-017-03883-7 28634348 PMC5478658

[39] Barzilay JI Farag YMK Durthaler JM . Albuminuria: An underappreciated risk factor for cardiovascular disease. J Am Heart Assoc. (2024) 13:e030131. doi: 10.1161/JAHA.123.030131 38214258 PMC10926810

[40] Tuttle KR . Cardiovascular implications of albuminuria. J Clin Hypertens (Greenwich). (2004) 6:13–7. doi: 10.1111/j.1524-6175.2004.4064.x 15538106 PMC8109333

[41] Maxwell SE Cole DA Mitchell MA . Bias in cross-sectional analyses of longitudinal mediation: partial and complete mediation under an autoregressive model. Multivariate Behav Res. (2011) 46:816–41. doi: 10.1080/00273171.2011.606716 26736047

[42] Pace C Smith-Gagen J Angermann J . Arsenic methylation capacity and metabolic syndrome in the 2013-2014 U.S. National Health and Nutrition Examination Survey (NHANES). Int J Environ Res Public Health. (2018) 15:168. doi: 10.3390/ijerph15010168 29361794 PMC5800267

[43] Abuawad A Spratlen MJ Parvez F Slavkovich V Ilievski V Lomax-Luu AM . Association between body mass index and arsenic methylation in three studies of Bangladeshi adults and adolescents. Environ Int. (2021) 149:106401. doi: 10.1016/j.envint.2021.106401 33549917 PMC7976732

[44] Sun X Briel M Walter SD Guyatt GH . Is a subgroup effect believable? Updating criteria to evaluate the credibility of subgroup analyses. BMJ. (2010) 340:c117. doi: 10.1136/bmj.c117 20354011

