# Supplementary material for: Arsenic species and lipid metabolism under low-to-moderate arsenic exposure: evidence from NHANES and a Chinese adult population
Source: Front Endocrinol (Lausanne). 2026 Aug 18;17:1919240. doi: 10.3389/fendo.2026.1919240 (PMC13529509; doi:10.3389/fendo.2026.1919240)
Supplement: Supplementary file 1 [file DataSheet1.zip › FIG S1-S2.docx]

**Figure S1. Flowchart of the participant’s selection from NHANES**

**Figure S2**. **Flowchart of the participant’s selection from the survey population of Zhaodong City, Heilongjiang Province**
