# Supplementary material for: Arsenic species and lipid metabolism under low-to-moderate arsenic exposure: evidence from NHANES and a Chinese adult population
Source: Front Endocrinol (Lausanne). 2026 Aug 18;17:1919240. doi: 10.3389/fendo.2026.1919240 (PMC13529509; doi:10.3389/fendo.2026.1919240)
Supplement: Supplementary file 1 [file DataSheet1.zip › FIG S3.docx]

**Figure S3. Restricted cubic spline analyses of the associations of urinary monomethylarsonic acid, As^III^, and As^V^ with TG, LDL-C, and non-HDL-C in survey-weighted NHANES and the Zhaodong population.** a2-c2, urinary monomethylarsonic acid in NHANES; a3-c3, urinary As ^III^ in NHANES; a4-c4, urinary As^V^ in NHANES; A2-C2, urinary monomethylarsonic acid in the Zhaodong population; A3-C3, urinary As^III^ in the Zhaodong population; A4-C4, urinary As^V^ in the Zhaodong population.
