# Supplementary material for: Arsenic species and lipid metabolism under low-to-moderate arsenic exposure: evidence from NHANES and a Chinese adult population
Source: Front Endocrinol (Lausanne). 2026 Aug 18;17:1919240. doi: 10.3389/fendo.2026.1919240 (PMC13529509; doi:10.3389/fendo.2026.1919240)
Supplement: Supplementary file 1 [file DataSheet1.zip › FIG S4.docx]

**Figure S4a. Subgroup analyses of the associations between urinary total arsenic and lipid metabolism indicators in survey-weighted NHANES and the Zhaodong population.** a–c represent TG, LDL-C, and non-HDL-C in NHANES, respectively; A–C represent TG, LDL-C, and non-HDL-C in the Zhaodong population, respectively. Points represent β coefficients and horizontal lines represent 95% confidence intervals. Estimates were derived from survey-weighted linear regression models. *P* for interaction was calculated using the fully adjusted model.

**Figure S4b. Subgroup analyses of the associations between urinary dimethylarsinic acid and lipid metabolism indicators in survey-weighted NHANES and the Zhaodong population.** a2–c2 represent TG, LDL-C, and non-HDL-C in NHANES, respectively; A2–C2 represent TG, LDL-C, and non-HDL-C in the Zhaodong population, respectively. Points represent β coefficients and horizontal lines represent 95% confidence intervals. Estimates were derived from survey-weighted linear regression models. *P* for interaction was calculated using the fully adjusted model.

**Figure S4c. Subgroup analyses of the associations between urinary monomethylarsonic acid and lipid metabolism indicators in survey-weighted NHANES and the Zhaodong population.** a1–c1 represent TG, LDL-C, and non-HDL-C in NHANES, respectively; A1–C1 represent TG, LDL-C, and non-HDL-C in the Zhaodong population, respectively. Points represent β coefficients and horizontal lines represent 95% confidence intervals. Estimates were derived from survey-weighted linear regression models. *P* for interaction was calculated using the fully adjusted model.

**Figure S4d. Subgroup analyses of the associations between urinary As^III^ acid and lipid metabolism indicators in survey-weighted NHANES and the Zhaodong population.** a3–c3 represent TG, LDL-C, and non-HDL-C in NHANES, respectively; A3–C3 represent TG, LDL-C, and non-HDL-C in the Zhaodong population, respectively. Points represent β coefficients and horizontal lines represent 95% confidence intervals. Estimates were derived from survey-weighted linear regression models. *P* for interaction was calculated using the fully adjusted model.

**Figure S4e. Subgroup analyses of the associations between urinary As^V^ and lipid metabolism indicators in survey-weighted NHANES and the Zhaodong population.** a4–c4 represent TG, LDL-C, and non-HDL-C in NHANES, respectively; A4–C4 represent TG, LDL-C, and non-HDL-C in the Zhaodong population, respectively. Points represent β coefficients and horizontal lines represent 95% confidence intervals. Estimates were derived from survey-weighted linear regression models. *P* for interaction was calculated using the fully adjusted model.
