# Supplementary material for: Arsenic species and lipid metabolism under low-to-moderate arsenic exposure: evidence from NHANES and a Chinese adult population
Source: Front Endocrinol (Lausanne). 2026 Aug 18;17:1919240. doi: 10.3389/fendo.2026.1919240 (PMC13529509; doi:10.3389/fendo.2026.1919240)
Supplement: Supplementary file 1 [file DataSheet1.zip › Table S1.docx]

**Table S1.** Limits of detection and substitution values for urinary total arsenic and arsenic species in NHANES and the Zhaodong population

| **Study population** | **Cycle** | **LOD, μg/L** | | | | | |  | **LOD/√2, μg/L** | | | | | |
| --- | --- | --- | --- | --- | --- | --- | --- | --- | --- | --- | --- | --- | --- | --- |
|  |  | **UTA** | **MMA** | **DMA** | **As^III^** | **As^V^** | **AsB** |  | **UTA** | **MMA** | **DMA** | **As^III^** | **As^V^** | **AsB** |
| **NHANES** | 2009-2010 | 0.74 | 0.90 | 1.70 | 1.20 | 1.00 | 0.40 |  | 0.52 | 0.64 | 1.20 | 0.85 | 0.71 | 0.28 |
|  | 2011-2012 | 1.25 | 0.89 | 1.80 | 0.48 | 0.87 | 1.19 |  | 0.88 | 0.63 | 1.27 | 0.34 | 0.62 | 0.84 |
|  | 2013-2014 | 0.26 | 0.20 | 1.91 | 0.12 | 0.79 | 1.16 |  | 0.18 | 0.14 | 1.35 | 0.08 | 0.56 | 0.82 |
|  | 2015-2016 | 0.26 | 0.20 | 1.91 | 0.12 | 0.79 | 1.16 |  | 0.18 | 0.14 | 1.35 | 0.08 | 0.56 | 0.82 |
|  | 2017-2020 | 0.23 | 0.20 | 1.91 | 0.12 | 0.79 | 1.16 |  | 0.16 | 0.14 | 1.35 | 0.08 | 0.56 | 0.82 |
| **Zhaodong**  **population** | - | 0.50 | 0.50 | 0.50 | 0.50 | 0.80 | - |  | 0.354 | 0.354 | 0.354 | 0.354 | 0.566 | - |
