# Supplementary material for: Arsenic species and lipid metabolism under low-to-moderate arsenic exposure: evidence from NHANES and a Chinese adult population
Source: Front Endocrinol (Lausanne). 2026 Aug 18;17:1919240. doi: 10.3389/fendo.2026.1919240 (PMC13529509; doi:10.3389/fendo.2026.1919240)
Supplement: Supplementary file 1 [file DataSheet1.zip › Table S10-S12.docx]

**Table S10. Baseline characteristics across quartiles of creatinine-corrected urinary MMA in the Zhaodong population**

| **Characteristics** | | **Total**  n=310 | **Q1** (< 1.25 ug/L cr)  n=78 | **Q2** (1.25-1.71 ug/L cr)  n=77 | **Q3** (1.71-2.83 ug/L cr)  n=77 | **Q4** (≥2.83 ug/L cr)  n=78 | ***P*** |
| --- | --- | --- | --- | --- | --- | --- | --- |
| Age (%) | |  |  |  |  |  | 0.899 |
| 18-39 | | 10 (3.23) | 3 (3.85) | 2 (2.60) | 1 (1.30) | 4 (5.13) |  |
| 39-60 | | 129 (41.61) | 33 (42.31) | 34 (44.16) | 31 (40.26) | 31 (39.74) |  |
| >60 | | 171 (55.16) | 42 (53.85) | 41 (53.25) | 45 (58.44) | 43 (55.13) |  |
| Sex (%) | |  |  |  |  |  | <0.001 |
| Female | | 190 (61.29) | 35 (44.87) | 43 (55.84) | 57 (74.03) | 55 (70.51) |  |
| Male | | 120 (38.71) | 43 (55.13) | 34 (44.16) | 20 (25.97) | 23 (29.49) |  |
| Nationality (%) |  | |  |  |  |  | 0.163 |
| Han Chinese | 292 (94.19) | | 77 (98.72) | 72 (93.51) | 70 (90.91) | 73 (93.59) |  |
| Other ethnic minorities | 18 (5.81) | | 1 (1.28) | 5 (6.49) | 7 (9.09) | 5 (6.41) |  |
| Education (%) |  | |  |  |  |  | 0.997 |
| Primary school and below | 207 (66.77) | | 53 (67.95) | 50 (64.94) | 52 (67.53) | 52 (66.67) |  |
| Junior high school | 79 (25.48) | | 19 (24.36) | 21 (27.27) | 20 (25.97) | 19 (24.36) |  |
| High school or above | 24 (7.74) | | 6 (7.69) | 6 (7.79) | 5 (6.49) | 7 (8.97) |  |
| Income (%) |  | |  |  |  |  | 0.489 |
| <20000 | 206 (66.45) | | 55 (70.51) | 49 (63.64) | 52 (67.53) | 50 (64.10) |  |
| 20000-60000 | 86 (27.74) | | 16 (20.51) | 25 (32.47) | 20 (25.97) | 25 (32.05) |  |
| >60000 | 18 (5.81) | | 7 (8.97) | 3 (3.90) | 5 (6.49) | 3 (3.85) |  |
| Smoke (%) |  | |  |  |  |  | 0.378 |
| Never | 211 (68.06) | | 49 (62.82) | 49 (63.64) | 52 (67.53) | 61 (78.21) |  |
| Ever | 17 (5.48) | | 4 (5.13) | 5 (6.49) | 4 (5.19) | 4 (5.13) |  |
| Now | 82 (26.45) | | 25 (32.05) | 23 (29.87) | 21 (27.27) | 13 (16.67) |  |
| Seafood (%) |  | |  |  |  |  | 0.624 |
| No | 171 (55.16) | | 45 (57.69) | 41 (53.25) | 41 (53.25) | 44 (56.41) |  |
| Once a day | 1 (0.32) | | 0 (0.00) | 1 (1.30) | 0 (0.00) | 0 (0.00) |  |
| At least once a week | 28 (9.03) | | 4 (5.13) | 10 (12.99) | 7 (9.09) | 7 (8.97) |  |
| At least once a month | 72 (23.23) | | 16 (20.51) | 20 (25.97) | 19 (24.68) | 17 (21.79) |  |
| At least once a year | 38 (12.26) | | 13 (16.67) | 5 (6.49) | 10 (12.99) | 10 (12.82) |  |
| Hypertension (%) |  | |  |  |  |  | 0.168 |
| No | 190 (61.29) | | 50 (64.10) | 54 (70.13) | 42 (54.55) | 44 (56.41) |  |
| Yes | 120 (38.71) | | 28 (35.90) | 23 (29.87) | 35 (45.45) | 34 (43.59) |  |
| eGFR (M (Q₁, Q₃)) | 98.51 (89.56,108.55) | | 101.27 (94.72,111.35) | 104.01 (94.60,114.11) | 102.56 (95.59,112.97) | 98.51 (89.56,108.55) | 0.073 |
| BMI (M (Q₁, Q₃)) | 24.80  (23.00, 26.80) | | 24.80 (23.33,26.93) | 24.80  (22.70,26.80) | 24.80  (23.50,26.80) | 24.80 (22.25,26.95) | 0.728 |

**Table S11. Baseline characteristics across quartiles of creatinine-corrected urinary As^III^ in the Zhaodong population**

| **Characteristics** | | **Total**  n=310 | **Total**  n=310 | **Q1** (< 3.77 ug/L cr)  n=78 | **Q2** (3.77-6.62 ug/L cr)  n=77 | **Q3** (6.62-11.92 ug/L cr)  n=77 | ***P*** |
| --- | --- | --- | --- | --- | --- | --- | --- |
| Age (%) | |  |  |  |  |  | 0.354 |
| 18-39 | | 10 (3.23) | 4 (5.13) | 4 (5.19) | 2 (2.60) | 0 (0.00) |  |
| 39-60 | | 129 (41.61) | 31 (39.74) | 36 (46.75) | 30 (38.96) | 32 (41.03) |  |
| >60 | | 171 (55.16) | 43 (55.13) | 37 (48.05) | 45 (58.44) | 46 (58.97) |  |
| Sex (%) | |  |  |  |  |  | 0.124 |
| Female | | 190 (61.29) | 39 (50.00) | 50 (64.94) | 49 (63.64) | 52 (66.67) |  |
| Male | | 120 (38.71) | 39 (50.00) | 27 (35.06) | 28 (36.36) | 26 (33.33) |  |
| Nationality (%) |  | |  |  |  |  | 0.752 |
| Han Chinese | 292 (94.19) | | 74 (94.87) | 74 (96.10) | 71 (92.21) | 73 (93.59) |  |
| Other ethnic minorities | 18 (5.81) | | 4 (5.13) | 3 (3.90) | 6 (7.79) | 5 (6.41) |  |
| Education (%) |  | |  |  |  |  | 0.546 |
| Primary school and below | 207 (66.77) | | 57 (73.08) | 54 (70.13) | 45 (58.44) | 51 (65.38) |  |
| Junior high school | 79 (25.48) | | 15 (19.23) | 18 (23.38) | 24 (31.17) | 22 (28.21) |  |
| High school or above | 24 (7.74) | | 6 (7.69) | 5 (6.49) | 8 (10.39) | 5 (6.41) |  |
| Income (%) |  | |  |  |  |  | 0.469 |
| <20000 | 206 (66.45) | | 50 (64.10) | 46 (59.74) | 52 (67.53) | 58 (74.36) |  |
| 20000-60000 | 86 (27.74) | | 24 (30.77) | 27 (35.06) | 19 (24.68) | 16 (20.51) |  |
| >60000 | 18 (5.81) | | 4 (5.13) | 4 (5.19) | 6 (7.79) | 4 (5.13) |  |
| Smoke (%) |  | |  |  |  |  | 0.845 |
| Never | 211 (68.06) | | 56 (71.79) | 55 (71.43) | 47 (61.04) | 53 (67.95) |  |
| Ever | 17 (5.48) | | 4 (5.13) | 4 (5.19) | 5 (6.49) | 4 (5.13) |  |
| Now | 82 (26.45) | | 18 (23.08) | 18 (23.38) | 25 (32.47) | 21 (26.92) |  |
| Seafood (%) |  | |  |  |  |  | 0.138 |
| No | 171 (55.16) | | 45 (57.69) | 47 (61.04) | 37 (48.05) | 42 (53.85) |  |
| Once a day | 1 (0.32) | | 1 (1.28) | 0 (0.00) | 0 (0.00) | 0 (0.00) |  |
| At least once a week | 28 (9.03) | | 9 (11.54) | 4 (5.19) | 10 (12.99) | 5 (6.41) |  |
| At least once a month | 72 (23.23) | | 15 (19.23) | 14 (18.18) | 17 (22.08) | 26 (33.33) |  |
| At least once a year | 38 (12.26) | | 8 (10.26) | 12 (15.58) | 13 (16.88) | 5 (6.41) |  |
| Hypertension (%) |  | |  |  |  |  | 0.798 |
| No | 190 (61.29) | | 45 (57.69) | 50 (64.94) | 46 (59.74) | 49 (62.82) |  |
| Yes | 120 (38.71) | | 33 (42.31) | 27 (35.06) | 31 (40.26) | 29 (37.18) |  |
| eGFR (M (Q₁, Q₃)) | 101.76  (93.24, 112.03) | | 100.74 (90.14,106.14) | 100.71 (89.98,111.90) | 102.79 (94.58,112.96) | 103.60 (95.77,113.61) | 0.163 |
| BMI (M (Q₁, Q₃)) | 24.80  (23.00, 26.80) | | 24.80 (22.75,25.88) | 24.80  (23.90,27.40) | 25.00  (23.20,27.10) | 24.80 (22.33,26.70) | 0.125 |

**Table S12. Baseline characteristics across quartiles of creatinine-corrected urinary As^V^ in the Zhaodong population**

| **Characteristics** | | **Total**  n=310 | **Q1** (< 1.92 ug/L cr)  n=78 | **Q2** (1.92-2.45 ug/L cr)  n=77 | **Q3** (2.45-3.73 ug/L cr)  n=77 | **Q4** (≥3.73 ug/L cr)  n=78 | ***P*** |
| --- | --- | --- | --- | --- | --- | --- | --- |
| Age (%) | |  |  |  |  |  | 0.876 |
| 18-39 | | 10 (3.23) | 3 (3.85) | 3 (3.90) | 2 (2.60) | 2 (2.56) |  |
| 39-60 | | 129 (41.61) | 33 (42.31) | 32 (41.56) | 36 (46.75) | 28 (35.90) |  |
| >60 | | 171 (55.16) | 42 (53.85) | 42 (54.55) | 39 (50.65) | 48 (61.54) |  |
| Sex (%) | |  |  |  |  |  | <0.001 |
| Female | | 190 (61.29) | 29 (37.18) | 41 (53.25) | 59 (76.62) | 61 (78.21) |  |
| Male | | 120 (38.71) | 49 (62.82) | 36 (46.75) | 18 (23.38) | 17 (21.79) |  |
| Nationality (%) |  | |  |  |  |  | 0.163 |
| Han Chinese | 292 (94.19) | | 77 (98.72) | 72 (93.51) | 70 (90.91) | 73 (93.59) |  |
| Other ethnic minorities | 18 (5.81) | | 1 (1.28) | 5 (6.49) | 7 (9.09) | 5 (6.41) |  |
| Education (%) |  | |  |  |  |  | 0.914 |
| Primary school and below | 207 (66.77) | | 50 (64.10) | 51 (66.23) | 56 (72.73) | 50 (64.10) |  |
| Junior high school | 79 (25.48) | | 22 (28.21) | 19 (24.68) | 16 (20.78) | 22 (28.21) |  |
| High school or above | 24 (7.74) | | 6 (7.69) | 7 (9.09) | 5 (6.49) | 6 (7.69) |  |
| Income (%) |  | |  |  |  |  | 0.404 |
| <20000 | 206 (66.45) | | 56 (71.79) | 51 (66.23) | 48 (62.34) | 51 (65.38) |  |
| 20000-60000 | 86 (27.74) | | 16 (20.51) | 22 (28.57) | 27 (35.06) | 21 (26.92) |  |
| >60000 | 18 (5.81) | | 6 (7.69) | 4 (5.19) | 2 (2.60) | 6 (7.69) |  |
| Smoke (%) |  | |  |  |  |  | 0.963 |
| Never | 211 (68.06) | | 50 (64.10) | 54 (70.13) | 51 (66.23) | 56 (71.79) |  |
| Ever | 17 (5.48) | | 5 (6.41) | 4 (5.19) | 4 (5.19) | 4 (5.13) |  |
| Now | 82 (26.45) | | 23 (29.49) | 19 (24.68) | 22 (28.57) | 18 (23.08) |  |
| Seafood (%) |  | |  |  |  |  | 0.394 |
| No | 171 (55.16) | | 45 (57.69) | 44 (57.14) | 44 (57.14) | 38 (48.72) |  |
| Once a day | 1 (0.32) | | 0 (0.00) | 0 (0.00) | 1 (1.30) | 0 (0.00) |  |
| At least once a week | 28 (9.03) | | 3 (3.85) | 10 (12.99) | 4 (5.19) | 11 (14.10) |  |
| At least once a month | 72 (23.23) | | 18 (23.08) | 16 (20.78) | 18 (23.38) | 20 (25.64) |  |
| At least once a year | 38 (12.26) | | 12 (15.38) | 7 (9.09) | 10 (12.99) | 9 (11.54) |  |
| Hypertension (%) |  | |  |  |  |  | 0.190 |
| No | 190 (61.29) | | 47 (60.26) | 55 (71.43) | 43 (55.84) | 45 (57.69) |  |
| Yes | 120 (38.71) | | 31 (39.74) | 22 (28.57) | 34 (44.16) | 33 (42.31) |  |
| eGFR (M (Q₁, Q₃)) | 101.76 (93.24, 112.03) | | 97.84 (87.35,106.24) | 100.72 (89.99,107.89) | 107.11 (100.71,115.82) | 103.03 (94.77,113.79) | <0.001 |
| BMI (M (Q₁, Q₃)) | 24.80 (23.00, 26.80) | | 24.80 (23.33,27.55) | 24.80  (22.70,26.70) | 24.80  (23.60,26.70) | 24.80 (22.75,26.80) | 0.875 |
