# Supplementary material for: Arsenic species and lipid metabolism under low-to-moderate arsenic exposure: evidence from NHANES and a Chinese adult population
Source: Front Endocrinol (Lausanne). 2026 Aug 18;17:1919240. doi: 10.3389/fendo.2026.1919240 (PMC13529509; doi:10.3389/fendo.2026.1919240)
Supplement: Supplementary file 1 [file DataSheet1.zip › Table S13-S17.docx]

**Table S13. Summary of P values for baseline characteristics across quartiles of creatinine-corrected urinary total arsenic and arsenic species in NHANES and the Zhaodong population**

| **Characteristics** | **NHANES, weighted** | | |  | **Zhaodong population** | | |
| --- | --- | --- | --- | --- | --- | --- | --- |
|  | **MMA** | **As^III^** | **As^V^** |  | **MMA** | **As^III^** | **As^V^** |
| Sex, (*P*) | <0.001 | <0.001 | <0.001 |  | <0.001 | 0.124 | <0.001 |
| Age, (*P*) | 0.490 | 0.261 | 0.005 |  | 0.899 | 0.354 | 0.876 |
| Race ^a^, (*P*)  Nationality ^b^, (*P*) | <0.001 | 0.012 | 0.005 |  | 0.163 | 0.752 | 0.163 |
| Education, (*P*) | 0.002 | 0.162 | 0.006 |  | 0.997 | 0.546 | 0.914 |
| PIR ^a^, (*P*)  Income ^b^, (*P*) | 0.017 | 0.038 | 0.011 |  | 0.489 | 0.469 | 0.404 |
| Cotinine ^a^, (*P*)  Smoking ^b^, (*P*) | 0.810 | 0.997 | 0.009 |  | 0.378 | 0.845 | 0.963 |
| Hypertension, (*P*) | 0.061 | 0.102 | 0.752 |  | 0.168 | 0.798 | 0.190 |
| Diabetes, (*P*) | 0.387 | 0.097 | 0.406 |  | - | - | - |
| BMI, (*P*) | <0.001 | <0.001 | <0.001 |  | 0.728 | 0.125 | 0.875 |
| EGFR, (*P*) | 0.268 | <0.001 | 0.020 |  | 0.073 | 0.163 | <0.001 |
| Urinary AsB ^a^, (*P*)  Seafood ^b^, (*P*) | <0.001 | <0.001 | 0.008 |  | 0.624 | 0.138 | 0.394 |
| Cycles, (*P*) | <0.001 | <0.001 | 0.024 |  | - | - | - |

Notes: a Indicates variables derived from NHANES; b Indicates variables derived from the Zhaodong population. Quartiles were defined separately according to creatinine-corrected urinary total arsenic and each arsenic species within each population. Creatinine-corrected urinary arsenic concentrations were calculated as urinary arsenic concentration (μg/L) × 100 / urinary creatinine (mg/dL), and expressed as μg/g creatinine.	MMA, monomethylarsonic acid; As^III^, arsenite; As^V^, arsenate.

**Table 14. Crude and adjusted linear associations between creatinine-corrected urinary total arsenic and arsenic species and lipid metabolism indicators in NHANES and the Zhaodong population**

| **Characteristics** | **Model** | **NHANES, weighted** | | |  | **Zhaodong population** | | |
| --- | --- | --- | --- | --- | --- | --- | --- | --- |
|  |  | **β (95%CI)** | ***P*** | ***P- for trend*** |  | **β (95%CI)** | ***P*** | ***P- for trend*** |
| **TG** | | | | | | | | |
| **MMA** | Model 1 | -1.11 (-9.61 ~ 7.39) | 0.799 | 0.402 |  | 55.62 (-18.53 ~ 129.77) | 0.143 | 0.114 |
|  | Model 2 | 6.15 (-3.44 ~ 15.75) | 0.214 | 0.604 |  | 52.60 (-19.70 ~ 124.89) | 0.155 | 0.168 |
| **As^III^** | Model 1 | 1.56 (-14.39 ~ 17.50) | 0.849 | 0.572 |  | 68.42 (-9.48 ~ 146.33) | 0.086 | 0.274 |
|  | Model 2 | 11.45 (-7.54 ~ 30.44) | 0.242 | 0.399 |  | 61.30 (-15.74 ~ 138.34) | 0.120 | 0.238 |
| **As^V^** | Model 1 | -3.03 (-11.45 ~ 5.39) | 0.483 | 0.243 |  | 353.84 (62.70 ~ 644.99) | 0.018 | 0.055 |
|  | Model 2 | 1.48 (-6.59 ~ 9.54) | 0.721 | 0.853 |  | 299.30 (10.69 ~ 587.90) | 0.043 | 0.303 |
| **LDL-C** | | | | | | | | |
| **MMA** | Model 1 | 0.06 (-3.39 ~ 3.52) | 0.971 | 0.462 |  | 6.47 (-14.01 ~ 26.95) | 0.536 | 0.754 |
|  | Model 2 | 0.33 (-2.61 ~ 3.27) | 0.826 | 0.425 |  | 4.98 (-15.63 ~ 25.58) | 0.636 | 0.600 |
| **As^III^** | Model 1 | -0.93 (-6.29 ~ 4.43) | 0.735 | 0.671 |  | 7.84 (-13.70 ~ 29.38) | 0.476 | 0.159 |
|  | Model 2 | -1.45 (-7.15 ~ 4.24) | 0.618 | 0.517 |  | -0.39 (-22.37 ~ 21.60) | 0.973 | 0.609 |
| **As^V^** | Model 1 | 0.84 (-2.99 ~ 4.66) | 0.670 | 0.470 |  | 4.72 (-76.19 ~ 85.64) | 0.909 | 0.677 |
|  | Model 2 | 0.47 (-3.42 ~ 4.36) | 0.814 | 0.267 |  | -18.76 (-101.31 ~ 63.80) | 0.656 | 0.471 |
| **Non-HDL-C** | | | | | | | | |
| **MMA** | Model 1 | 0.61 (-1.52 ~ 2.74) | 0.576 | 0.918 |  | 17.99 (-7.96 ~ 43.93) | 0.175 | 0.795 |
|  | Model 2 | 0.25 (-2.32 ~ 2.82) | 0.850 | 0.267 |  | 15.40 (-10.21 ~ 41.02) | 0.239 | 0.782 |
| **As^III^** | Model 1 | 0.78 (-1.82 ~ 3.38) | 0.559 | 0.774 |  | 20.08 (-7.21 ~ 47.37) | 0.150 | 0.142 |
|  | Model 2 | -0.32 (-3.59 ~ 2.96) | 0.849 | 0.427 |  | 10.78 (-16.57 ~ 38.13) | 0.440 | 0.416 |
| **As^V^** | Model 1 | -1.52 (-4.15 ~ 1.10) | 0.259 | 0.050 |  | 29.80 (-72.91 ~ 132.51) | 0.570 | 0.662 |
|  | Model 2 | -0.52 (-3.09 ~ 2.04) | 0.690 | 0.168 |  | -12.32 (-115.17 ~ 90.52) | 0.814 | 0.231 |

Notes: NHANES: Model 1: Unadjusted (Crude); Model 2: Adjust (Sex, Age, Race, Education, INDFMPIR, Cotinine, Hypertension, BMXBMI, EGFR, Urinary AsB, Data release number). Zhaodong population: Model 1: Unadjusted (Crude); Model 2: Adjust (Age, Sex, Nationality, Education, Income, BMI, eGFR, Smoke, Seafood, Hypertension). MMA, monomethylarsonic acid; As^III^, arsenite; As^V^, arsenate.

**Table S15. Crude and adjusted logistic associations between quartiles of creatinine-corrected urinary total arsenic and arsenic species and lipid metabolism abnormalities in NHANES and the Zhaodong population**

| **Characteristics** | | **Quartile** | **NHANES, weighted OR (95% CI)** | |  | **Zhaodong population OR (95% CI)** | |
| --- | --- | --- | --- | --- | --- | --- | --- |
|  |  |  | Model 1 | Model 2 |  | Model 1 | Model 2 |
| **TG** | | | | | | | |
|  | **MMA** | Q1 | 1.00 (Reference) | 1.00 (Reference) |  | 1.00 (Reference) | 1.00 (Reference) |
|  |  | Q2 | 1.19 (0.68 ~ 2.08) | 1.45 (0.75 ~ 2.80) |  | 1.33 (0.71 ~ 2.50) | 1.32 (0.66 ~ 2.63) |
|  |  | Q3 | 1.01 (0.57 ~ 1.80) | 1.24 (0.63 ~ 2.47) |  | 1.08 (0.57 ~ 2.03) | 0.81 (0.40 ~ 1.64) |
|  |  | Q4 | 0.81 (0.47 ~ 1.37) | 1.19 (0.55 ~ 2.57) |  | 1.29 (0.69 ~ 2.43) | 1.16 (0.57 ~ 2.35) |
|  | **As^III^** | Q1 | 1.00 (Reference) | 1.00 (Reference) |  | 1.00 (Reference) | 1.00 (Reference) |
|  |  | Q2 | 0.91 (0.46 ~ 1.80) | 0.93 (0.44 ~ 1.96) |  | 1.92 (1.01 ~ 3.65)* | 1.99 (0.98 ~ 4.03) |
|  |  | Q3 | 0.79 (0.49 ~ 1.27) | 0.72 (0.38 ~ 1.37) |  | 1.73 (0.91 ~ 3.29) | 1.72 (0.84 ~ 3.52) |
|  |  | Q4 | 0.87 (0.54 ~ 1.40) | 1.30 (0.61 ~ 2.77) |  | 1.61 (0.85 ~ 3.04) | 1.72 (0.84 ~ 3.50) |
|  | **As^V^** | Q1 | 1.00 (Reference) | 1.00 (Reference) |  | 1.00 (Reference) | 1.00 (Reference) |
|  |  | Q2 | 0.63 (0.38 ~ 1.03) | 0.70 (0.41 ~ 1.20) |  | 1.14 (0.60 ~ 2.14) | 1.14 (0.57 ~ 2.30) |
|  |  | Q3 | 0.87 (0.46 ~ 1.65) | 0.94 (0.46 ~ 1.92) |  | 1.26 (0.67 ~ 2.38) | 0.94 (0.45 ~ 1.94) |
|  |  | Q4 | 0.79 (0.47 ~ 1.32) | 1.07 (0.58 ~ 1.96) |  | 1.59 (0.85 ~ 2.99) | 1.24 (0.60 ~ 2.56) |
| **LDL-C** | | | | | | | |
|  | **MMA** | Q1 | 1.00 (Reference) | 1.00 (Reference) |  | 1.00 (Reference) | 1.00 (Reference) |
|  |  | Q2 | 1.23 (0.57 ~ 2.64) | 1.14 (0.56 ~ 2.33) |  | 2.34 (0.98 ~ 5.60) | 2.21 (0.89 ~ 5.52) |
|  |  | Q3 | 1.10 (0.63 ~ 1.94) | 1.05 (0.59 ~ 1.85) |  | 2.01 (0.83 ~ 4.88) | 1.47 (0.58 ~ 3.75) |
|  |  | Q4 | 0.80 (0.41 ~ 1.57) | 0.86 (0.42 ~ 1.76) |  | 1.83 (0.75 ~ 4.46) | 1.39 (0.54 ~ 3.60) |
|  | **As^III^** | Q1 | 1.00 (Reference) | 1.00 (Reference) |  | 1.00 (Reference) | 1.00 (Reference) |
|  |  | Q2 | 0.99 (0.58 ~ 1.70) | 0.91 (0.52 ~ 1.60) |  | 2.07 (0.89 ~ 4.84) | 2.30 (0.95 ~ 5.62) |
|  |  | Q3 | 0.80 (0.47 ~ 1.38) | 0.65 (0.35 ~ 1.19) |  | 1.38 (0.57 ~ 3.37) | 1.21 (0.48 ~ 3.10) |
|  |  | Q4 | 0.76 (0.41 ~ 1.40) | 0.69 (0.33 ~ 1.46) |  | 1.90 (0.81 ~ 4.45) | 1.54 (0.63 ~ 3.79) |
|  | **As^V^** | Q1 | 1.00 (Reference) | 1.00 (Reference) |  | 1.00 (Reference) | 1.00 (Reference) |
|  |  | Q2 | 0.68 (0.42 ~ 1.09) | 0.71 (0.42 ~ 1.19) |  | 1.02 (0.43 ~ 2.42) | 0.92 (0.37 ~ 2.30) |
|  |  | Q3 | 0.85 (0.50 ~ 1.45) | 0.85 (0.45 ~ 1.61) |  | 1.80 (0.81 ~ 4.03) | 1.33 (0.55 ~ 3.23) |
|  |  | Q4 | 0.74 (0.45 ~ 1.22) | 0.76 (0.43 ~ 1.34) |  | 1.31 (0.57 ~ 3.01) | 0.91 (0.37 ~ 2.27) |
| **Non-HDL-C** | | | | | | | |
|  | **MMA** | Q1 | 1.00 (Reference) | 1.00 (Reference) |  | 1.00 (Reference) | 1.00 (Reference) |
|  |  | Q2 | 1.12 (0.81 ~ 1.56) | 1.04 (0.72 ~ 1.50) |  | 1.20 (0.59 ~ 2.45) | 1.12 (0.52 ~ 2.39) |
|  |  | Q3 | 1.08 (0.83 ~ 1.40) | 1.06 (0.77 ~ 1.46) |  | 1.29 (0.63 ~ 2.65) | 1.00 (0.46 ~ 2.19) |
|  |  | Q4 | 0.97 (0.73 ~ 1.27) | 0.81 (0.54 ~ 1.22) |  | 1.07 (0.53 ~ 2.15) | 1.00 (0.46 ~ 2.14) |
|  | **As^III^** | Q1 | 1.00 (Reference) | 1.00 (Reference) |  | 1.00 (Reference) | 1.00 (Reference) |
|  |  | Q2 | 0.89 (0.55 ~ 1.46) | 0.80 (0.47 ~ 1.37) |  | 2.16 (1.08 ~ 4.34)* | 2.16 (1.08 ~ 4.34) |
|  |  | Q3 | 0.92 (0.61 ~ 1.41) | 0.75 (0.46 ~ 1.22) |  | 2.97 (1.42 ~ 6.19)** | 2.97 (1.42 ~ 6.19)* |
|  |  | Q4 | 0.92 (0.62 ~ 1.36) | 0.69 (0.41 ~ 1.15) |  | 2.37 (1.17 ~ 4.78)* | 2.37 (1.17 ~ 4.78) |
|  | **As^V^** | Q1 | 1.00 (Reference) | 1.00 (Reference) |  | 1.00 (Reference) | 1.00 (Reference) |
|  |  | Q2 | 1.01 (0.78 ~ 1.31) | 1.02 (0.78 ~ 1.35) |  | 1.04 (0.53 ~ 2.07) | 0.93 (0.45 ~ 1.94) |
|  |  | Q3 | 1.04 (0.76 ~ 1.42) | 1.07 (0.74 ~ 1.55) |  | 1.84 (0.88 ~ 3.85) | 1.29 (0.57 ~ 2.92) |
|  |  | Q4 | 0.72 (0.51 ~ 1.02) | 0.82 (0.59 ~ 1.14) |  | 1.48 (0.73 ~ 3.02) | 1.07 (0.48 ~ 2.38) |

Notes: NHANES: Model 1: Unadjusted (Crude); Model 2: Adjust (Sex, Age, Race, Education, INDFMPIR, Cotinine, Hypertension, BMI, EGFR, Urinary AsB, Data release number). Zhaodong population: Model 1: Unadjusted (Crude); Model 2: Adjust (Age, Sex, Nationality, Education, Income, BMI, eGFR, Smoke, Seafood, Hypertension). Signif. codes: ‘***’ 0.001； ‘**’ 0.01； ‘*’ 0.05. MMA, monomethylarsonic acid; As^III^, arsenite; As^V^, arsenate.

**Table 16. Crude and adjusted linear associations of creatinine-corrected urinary total arsenic and arsenic species with UACR, and associations of UACR with lipid metabolism indicators in NHANES and the Zhaodong population.**

| **Characteristics** | **Model** | **NHANES, weighted** | | |  | **Zhaodong population** | | |
| --- | --- | --- | --- | --- | --- | --- | --- | --- |
|  |  | **β (95%CI)** | ***P*** | ***P- for trend*** |  | **β (95%CI)** | ***P*** | ***P- for trend*** |
| **MMA to UACR** | | | | | | | | |
|  | Model 1 | -3.67 (-12.13 ~ 4.78) | 0.397 | 0.351 |  | 0.49 (-32.99 ~ 33.97) | 0.977 | 0.328 |
|  | Model 2 | 11.17 (1.81 ~ 20.53) | 0.023 | 0.065 |  | -9.16 (-40.89 ~ 22.57) | 0.572 | 0.560 |
| **As^III^ to UACR** | | | | | | | | |
|  | Model 1 | -8.40 (-16.19 ~ -0.61) | 0.037 | 0.085 |  | -8.91 (-44.12 ~ 26.29) | 0.620 | 0.697 |
|  | Model 2 | 8.21 (0.18 ~ 16.23) | 0.049 | 0.107 |  | -24.77 (-58.50 ~ 8.97) | 0.151 | 0.310 |
| **As^V^ to UACR** | | | | | | | | |
|  | Model 1 | -0.58 (-5.09 ~ 3.94) | 0.803 | 0.756 |  | 40.80 (-91.31 ~ 172.92) | 0.545 | 0.099 |
|  | Model 2 | 10.43 (1.99 ~ 18.87) | 0.018 | 0.011 |  | -4.84 (-132.03 ~ 122.35) | 0.941 | 0.832 |

Notes: NHANES: Model 1: Unadjusted (Crude); Model 2: Adjust (Sex, Age, Race, Education, INDFMPIR, Cotinine, Hypertension, BMXBMI, EGFR, Urinary AsB, Data release number). Zhaodong population: Model 1: Unadjusted (Crude); Model 2: Adjust (Age, Sex, Nationality, Education, Income, BMI, eGFR, Smoke, Seafood, Hypertension). MMA, monomethylarsonic acid; As^III^, arsenite; As^V^, arsenate.

**Table 17. Mediation analysis of UACR in the associations between urinary total arsenic, arsenic species, and lipid metabolism indicators in the NHANES and Zhaodong populations**

| **Exposure** | **Mediator** | **Outcome** | **ACME**  **95%CI** | ***P*** | **ADE**  **95%CI** | ***P*** | **Total**  **Effect**  **95%CI** | ***P*** | **Proportion**  **of**  **mediation (%)** |
| --- | --- | --- | --- | --- | --- | --- | --- | --- | --- |
| **NHANES, weighted** | | | | | | | | | |
| **MMA** | UACR | TG | 0.22  (-0.27 ~ 0.98) | 0.466 | 6.64  (-2.25 ~ 15.61) | 0.158 | 6.87  (-2.17 ~ 15.82) | 0.144 | 2.26 |
|  |  | LDL-C | -0.07  (-0.17 ~ -0.00) | 0.046 | 0.47  (-2.38 ~ 3.41) | 0.736 | 0.40  (-2.45 ~ 3.38) | 0.790 | -1.90 |
|  |  | Non-HDL-C | 0.04  (-0.11 ~ 0.23) | 0.574 | 0.19  (-2.35 ~ 2.78) | 0.912 | 0.23  (-2.31 ~ 2.79) | 0.876 | 0.32 |
| **As^III^** | UACR | TG | 0.26  (-0.30 ~ 1.13) | 0.432 | 12.18  (-6.03 ~ 31.27) | 0.202 | 12.44  (-5.96 ~ 31.35) | 0.194 | 1.33 |
|  |  | LDL-C | -0.09  (-0.22 ~ 0.01) | 0.092 | -1.28  (-7.04 ~ 4.45) | 0.682 | -1.37  (-7.13 ~ 4.35) | 0.666 | 1.60 |
|  |  | Non-HDL-C | 0.03  (-0.08 ~ 0.18) | 0.572 | -0.39  (-3.65 ~ 3.01) | 0.770 | -0.35  (-3.59 ~ 3.04) | 0.790 | -0.31 |
| **As^V^** | UACR | TG | 0.31  (-0.34 ~ 1.27) | 0.384 | 2.54  (-5.87 ~ 10.73) | 0.528 | 2.85  (-5.56 ~ 11.18) | 0.510 | 3.77 |
|  |  | LDL-C | -0.12  (-0.30 ~ 0.01) | 0.074 | 0.68  (-3.19 ~ 4.58) | 0.732 | 0.56  (-3.27 ~ 4.45) | 0.772 | -2.46 |
|  |  | Non-HDL-C | 0.04  (-0.10 ~ 0.21) | 0.540 | -0.56  (-3.10 ~ 1.92) | 0.654 | -0.52  (-3.09 ~ 2.00) | 0.678 | 0.35 |
| **Zhaodong population** | | | | | | | | | |
| **MMA** | UACR | TG | -4.20  (-22.58 ~ 9.93) | 0.580 | 56.76  (-15.74 ~ 132.7) | 0.112 | 52.56  (-20.36 ~ 127.4) | 0.150 | -5.05 |
|  |  | LDL-C | -0.086  (-1.87 ~ 1.23) | 0.966 | 5.09  (-15.99 ~ 27.16) | 0.632 | 5.005  (-16.08 ~ 27.19) | 0.636 | 0.013 |
|  |  | Non-HDL-C | -0.56  (-3.93 ~1.62) | 0.676 | 15.98  (-10.15 ~ 43.34) | 0.220 | 15.42  (-10.28 ~ 42.46) | 0.238 | -1.22 |
| **As^III^** | UACR | TG | -11.53  (-32.84 ~ 3.07) | 0.128 | 72.71  (-3.86 ~ 154.26) | 0.056 | 61.18  (-17.14 ~ 141.9) | 0.126 | -14.42 |
|  |  | LDL-C | -0.22  (-2.91 ~ 1.94) | 0.824 | -0.16  (-22.47 ~ 23.60) | 0.976 | -0.38  (-22.30 ~ 23.01) | 0.966 | 0.213 |
|  |  | Non-HDL-C | -1.53  (-6.26 ~ 1.13) | 0.282 | 12.30  (-15.38 ~ 41.78) | 0.370 | 10.77  (-16.98 ~ 39.31) | 0.426 | -4.541 |
| **As^V^** | UACR | TG | -2.54  (-72.00 ~ 55.21) | 0.938 | 301.64  (17.85 ~ 604.33) | 0.044 | 299.10  (9.19 ~ 603.20) | 0.046 | -0.671 |
|  |  | LDL-C | -0.026  (-5.74 ~ 5.01) | 0.992 | -18.65  (-101.4 ~ 69.6) | 0.664 | -18.68  (-102.7 ~ 68.7) | 0.668 | 0.038 |
|  |  | Non-HDL-C | -0.31  (-12.19 ~ 9.32) | 0.970 | -11.97  (-114.8 ~ 97.74) | 0.808 | -12.28  (-117.4 ~ 96.38) | 0.800 | 0.011 |

Notes: NHANES: Model 1: Unadjusted (Crude); Model 2: Adjust (Sex, Age, Race, Education, INDFMPIR, Cotinine, Hypertension, BMI, EGFR, Urinary AsB, Data release number). Zhaodong population: Model 1: Unadjusted (Crude); Model 2: Adjust (Age, Sex, Nationality, Education, Income, BMI, eGFR, Smoke, Seafood, Hypertension). MMA, monomethylarsonic acid; As^III^, arsenite; As^V^, arsenate.
