# Supplementary material for: Arsenic species and lipid metabolism under low-to-moderate arsenic exposure: evidence from NHANES and a Chinese adult population
Source: Front Endocrinol (Lausanne). 2026 Aug 18;17:1919240. doi: 10.3389/fendo.2026.1919240 (PMC13529509; doi:10.3389/fendo.2026.1919240)
Supplement: Supplementary file 1 [file DataSheet1.zip › Table S2-S3.docx]

**Table S2. Baseline characteristics across quartiles of creatinine-corrected urinary total arsenic in NHANES**

| **Characteristics** | **Total**  N= 52612536  n= 2104 | **Q1**  (< 2.93 ug/L cr)  N= 13152488  n= 524 | **Q2**  (2.93-4.08 ug/L cr)  N= 13148012  n= 467 | **Q3**  (4.08-5.95 ug/L cr)  N= 13142234  n= 512 | **Q4**  (**≥** 5.95 ug/L cr)  N= 13169802  n= 601 | ***P*** |
| --- | --- | --- | --- | --- | --- | --- |
|  |  |  |  |  |  |  |
| Sex, n (%) |  |  |  |  |  | <0.001 |
| Female | 857 (37.61) | 277 (49.62) | 202 (41.46) | 199 (32.92) | 179 (26.45) |  |
| Male | 1247 (62.39) | 247 (50.38) | 265 (58.54) | 313 (67.08) | 422 (73.55) |  |
| Age, n (%) |  |  |  |  |  | 0.050 |
| 20-39 | 495 (26.67) | 144 (30.95) | 127 (29.92) | 118 (25.34) | 106 (20.48) |  |
| 40-60 | 766 (41.81) | 196 (42.83) | 159 (39.99) | 189 (41.51) | 222 (42.90) |  |
| > 60 | 843 (31.52) | 184 (26.22) | 181 (30.09) | 205 (33.15) | 273 (36.62) |  |
| Race, n (%) |  |  |  |  |  | <0.001 |
| Mexican American | 271 (5.84) | 33 (3.85) | 55 (5.61) | 83 (6.74) | 100 (7.15) |  |
| Other Hispanic | 197 (3.96) | 22 (1.71) | 25 (2.50) | 34 (2.78) | 116 (8.83) |  |
| Non-Hispanic White | 1106 (77.63) | 284 (77.59) | 281 (81.52) | 284 (78.94) | 257 (72.49) |  |
| Non-Hispanic Black | 359 (7.46) | 147 (11.77) | 81 (6.54) | 72 (6.79) | 59 (4.74) |  |
| Other Race - Including Multi-Racial | 171 (5.11) | 38 (5.08) | 25 (3.83) | 39 (4.75) | 69 (6.79) |  |
| Education, n (%) |  |  |  |  |  | 0.011 |
| Less Than 9th Grade | 205 (5.04) | 39 (5.27) | 37 (4.02) | 39 (3.10) | 90 (7.76) |  |
| 9-11th Grade (Includes 12th grade with no diploma) | 289 (9.33) | 84 (12.04) | 52 (6.76) | 72 (9.67) | 81 (8.87) |  |
| High School Grad/GED or Equivalent | 501 (24.58) | 140 (27.35) | 128 (29.11) | 119 (22.25) | 114 (19.62) |  |
| Some College or AA degree | 686 (33.96) | 169 (31.48) | 162 (35.10) | 171 (36.33) | 184 (32.93) |  |
| College Graduate or above | 423 (27.09) | 92 (23.86) | 88 (25.01) | 111 (28.65) | 132 (30.81) |  |
| PIR, n (%) |  |  |  |  |  | 0.133 |
| ≤1.3 | 716 (22.11) | 193 (23.85) | 155 (19.55) | 162 (23.00) | 206 (22.04) |  |
| 1.3-3.5 | 794 (37.43) | 202 (39.55) | 178 (42.82) | 201 (35.55) | 213 (31.78) |  |
| ＞3.5 | 594 (40.47) | 129 (36.60) | 134 (37.63) | 149 (41.45) | 182 (46.18) |  |
| Cotinine, n (%) |  |  |  |  |  | 0.003 |
| < 0.05 | 1136 (57.00) | 223 (48.47) | 229 (56.48) | 300 (59.88) | 384 (63.15) |  |
| 0.05-10 | 410 (16.53) | 130 (20.82) | 97 (15.74) | 81 (13.80) | 102 (15.73) |  |
| > 10 | 558 (26.48) | 171 (30.70) | 141 (27.77) | 131 (26.32) | 115 (21.12) |  |
| Hypertension, n (%) |  |  |  |  |  | 0.624 |
| No | 939 (51.62) | 220 (49.50) | 217 (54.58) | 229 (49.95) | 273 (52.43) |  |
| Yes | 1165 (48.38) | 304 (50.50) | 250 (45.42) | 283 (50.05) | 328 (47.57) |  |
| Diabetes, n (%) |  |  |  |  |  | 0.384 |
| No | 1558 (79.36) | 398 (78.65) | 345 (76.71) | 377 (79.29) | 438 (82.78) |  |
| Yes | 546 (20.64) | 126 (21.35) | 122 (23.29) | 135 (20.71) | 163 (17.22) |  |
| BMI, Mean (SE) | 29.95 (0.23) | 31.51 (0.39) | 30.54 (0.37) | 29.81 (0.46) | 27.95 (0.33) | <0.001 |
| EGFR, Mean (SE) | 92.47 (0.61) | 89.16 (1.07) | 94.18 (0.94) | 92.52 (1.16) | 94.03 (1.11) | 0.003 |
| Urinary AsB, Mean (SE) | 0.76 (0.01) | 0.78 (0.01) | 0.77 (0.01) | 0.75 (0.01) | 0.76 (0.01) | 0.084 |
| Data release number, n (%) | |  |  |  |  | <0.001 |
| 2009-2010 | 442 (15.51) | 57 (8.25) | 88 (12.71) | 123 (19.21) | 174 (21.85) |  |
| 2011-2012 | 344 (18.35) | 84 (15.38) | 74 (17.56) | 86 (21.14) | 100 (19.31) |  |
| 2013-2014 | 384 (18.00) | 93 (18.07) | 89 (17.97) | 93 (17.63) | 109 (18.34) |  |
| 2015-2016 | 374 (18.97) | 98 (21.53) | 85 (20.67) | 98 (17.21) | 93 (16.47) |  |
| 2017-2020 | 560 (29.17) | 192 (36.76) | 131 (31.10) | 112 (24.81) | 125 (24.03) |  |

**Table S3. Baseline characteristics across quartiles of creatinine-corrected urinary DMA in NHANES**

| **Characteristics** | **Total**  N= 52612536  n= 2104 | **Q1**  (<1.96 ug/L cr)  N= 13034792  n= 526 | **Q2**  (1.96-2.98 ug/L cr)  N= 13254142  n= 507 | **Q3**  (2.98-4.54 ug/L cr)  N= 13165898  n= 534 | **Q4**  (**≥** 4.54 ug/L cr)  N= 13157704  n= 537 | ***P*** |
| --- | --- | --- | --- | --- | --- | --- |
|  |  |  |  |  |  |  |
| Sex, n (%) |  |  |  |  |  | <0.001 |
| Female | 857 (37.61) | 303 (55.30) | 222 (42.47) | 199 (31.12) | 133 (21.69) |  |
| Male | 1247 (62.39) | 223 (44.70) | 285 (57.53) | 335 (68.88) | 404 (78.31) |  |
| Age, n (%) |  |  |  |  |  | 0.001 |
| 20-39 | 495 (26.67) | 162 (35.55) | 128 (28.05) | 103 (20.84) | 102 (22.32) |  |
| 40-60 | 766 (41.81) | 188 (40.12) | 185 (41.24) | 190 (41.42) | 203 (44.45) |  |
| > 60 | 843 (31.52) | 176 (24.33) | 194 (30.71) | 241 (37.74) | 232 (33.23) |  |
| Race, n (%) |  |  |  |  |  | <0.001 |
| Mexican American | 271 (5.84) | 44 (4.78) | 58 (5.30) | 100 (8.00) | 69 (5.25) |  |
| Other Hispanic | 197 (3.96) | 20 (1.83) | 23 (1.89) | 48 (3.85) | 106 (8.27) |  |
| Non-Hispanic White | 1106 (77.63) | 282 (77.95) | 309 (80.87) | 264 (75.53) | 251 (76.15) |  |
| Non-Hispanic Black | 359 (7.46) | 153 (12.52) | 88 (7.02) | 74 (6.68) | 44 (3.67) |  |
| Other Race - Including Multi-Racial | 171 (5.11) | 27 (2.92) | 29 (4.91) | 48 (5.95) | 67 (6.66) |  |
| Education, n (%) |  |  |  |  |  | 0.023 |
| Less Than 9th Grade | 205 (5.04) | 40 (5.98) | 37 (3.35) | 55 (4.44) | 73 (6.41) |  |
| 9-11th Grade (Includes 12th grade with no diploma) | 289 (9.33) | 87 (10.71) | 62 (8.83) | 72 (9.28) | 68 (8.54) |  |
| High School Grad/GED or Equivalent | 501 (24.58) | 148 (29.67) | 127 (24.86) | 130 (26.37) | 96 (17.48) |  |
| Some College or AA degree | 686 (33.96) | 162 (31.14) | 175 (35.01) | 176 (34.58) | 173 (35.06) |  |
| College Graduate or above | 423 (27.09) | 89 (22.50) | 106 (27.96) | 101 (25.33) | 127 (32.51) |  |
| PIR, n (%) |  |  |  |  |  | 0.126 |
| ≤1.3 | 716 (22.11) | 195 (24.68) | 167 (21.16) | 173 (22.35) | 181 (20.27) |  |
| 1.3-3.5 | 794 (37.43) | 205 (39.59) | 190 (38.67) | 214 (39.15) | 185 (32.31) |  |
| ＞3.5 | 594 (40.47) | 126 (35.73) | 150 (40.17) | 147 (38.50) | 171 (47.42) |  |
| Cotinine, n (%) |  |  |  |  |  | <0.001 |
| < 0.05 | 1136 (57.00) | 222 (47.71) | 245 (52.70) | 320 (62.98) | 349 (64.54) |  |
| 0.05-10 | 410 (16.53) | 131 (21.02) | 106 (17.73) | 84 (11.74) | 89 (15.66) |  |
| > 10 | 558 (26.48) | 173 (31.27) | 156 (29.57) | 130 (25.28) | 99 (19.80) |  |
| Hypertension, n (%) |  |  |  |  |  | 0.540 |
| No | 939 (51.62) | 241 (54.87) | 223 (51.34) | 225 (47.83) | 250 (52.45) |  |
| Yes | 1165 (48.38) | 285 (45.13) | 284 (48.66) | 309 (52.17) | 287 (47.55) |  |
| Diabetes, n (%) |  |  |  |  |  | 0.558 |
| No | 1558 (79.36) | 398 (79.22) | 376 (79.94) | 390 (76.64) | 394 (81.64) |  |
| Yes | 546 (20.64) | 546 (20.64) | 546 (20.64) | 546 (20.64) | 546 (20.64) |  |
| BMI, Mean (SE) | 29.95 (0.23) | 30.62 (0.34) | 30.75 (0.43) | 30.20 (0.48) | 28.24 (0.46) | <0.001 |
| EGFR, Mean (SE) | 92.47 (0.61) | 91.65 (1.25) | 91.86 (1.04) | 92.02 (1.06) | 94.36 (1.17) | 0.322 |
| Urinary AsB, Mean (SE) | 0.76 (0.01) | 0.76 (0.01) | 0.76 (0.01) | 0.76 (0.01) | 0.78 (0.01) | 0.364 |
| Data release number, n (%) | |  |  |  |  | 0.277 |
| 2009-2010 | 442 (15.51) | 101 (13.49) | 115 (17.41) | 120 (17.13) | 106 (13.97) |  |
| 2011-2012 | 344 (18.35) | 78 (17.10) | 82 (18.01) | 89 (19.22) | 95 (19.04) |  |
| 2013-2014 | 384 (18.00) | 74 (14.39) | 103 (20.30) | 106 (17.97) | 101 (19.30) |  |
| 2015-2016 | 374 (18.97) | 94 (19.57) | 89 (19.88) | 95 (19.61) | 96 (16.83) |  |
| 2017-2020 | 560 (29.17) | 179 (35.44) | 118 (24.40) | 124 (26.08) | 139 (30.87) |  |
