# Supplementary material for: Arsenic species and lipid metabolism under low-to-moderate arsenic exposure: evidence from NHANES and a Chinese adult population
Source: Front Endocrinol (Lausanne). 2026 Aug 18;17:1919240. doi: 10.3389/fendo.2026.1919240 (PMC13529509; doi:10.3389/fendo.2026.1919240)
Supplement: Supplementary file 1 [file DataSheet1.zip › Table S4-S5.docx]

**Table S4. Baseline characteristics across quartiles of creatinine-corrected urinary total arsenic in the Zhaodong population**

| **Characteristics** | | **Total**  n=310 | **Q1** (< 36.62 ug/L cr)  n=78 | **Q2** (36.62-57.09 ug/L cr)  n=77 | **Q3** (57.09-82.85 ug/L cr)  n=77 | **Q4** (≥ 82.85 ug/L cr)  n=78 | ***P*** |
| --- | --- | --- | --- | --- | --- | --- | --- |
| Age (%) | |  |  |  |  |  | 0.509 |
| 18-39 | | 10 (3.23) | 5 (6.41) | 1 (1.30) | 1 (1.30) | 3 (3.85) |  |
| 39-60 | | 129 (41.61) | 34 (43.59) | 31 (40.26) | 35 (45.45) | 29 (37.18) |  |
| >60 | | 171 (55.16) | 39 (50.00) | 45 (58.44) | 41 (53.25) | 46 (58.97) |  |
| Sex (%) | |  |  |  |  |  | 0.082 |
| Female | | 190 (61.29) | 44 (56.41) | 41 (53.25) | 49 (63.64) | 56 (71.79) |  |
| Male | | 120 (38.71) | 34 (43.59) | 36 (46.75) | 28 (36.36) | 22 (28.21) |  |
| Nationality (%) |  | |  |  |  |  | 0.382 |
| Han Chinese | 292 (94.19) | | 76 (97.44) | 72 (93.51) | 73 (94.81) | 71 (91.03) |  |
| Other ethnic minorities | 18 (5.81) | | 2 (2.56) | 5 (6.49) | 4 (5.19) | 7 (8.97) |  |
| Education (%) |  | |  |  |  |  | 0.841 |
| Primary school and below | 207 (66.77) | | 51 (65.38) | 53 (68.83) | 50 (64.94) | 53 (67.95) |  |
| Junior high school | 79 (25.48) | | 20 (25.64) | 19 (24.68) | 23 (29.87) | 17 (21.79) |  |
| High school or above | 24 (7.74) | | 7 (8.97) | 5 (6.49) | 4 (5.19) | 8 (10.26) |  |
| Income (%) |  | |  |  |  |  | 0.342 |
| <20000 | 206 (66.45) | | 47 (60.26) | 56 (72.73) | 55 (71.43) | 48 (61.54) |  |
| 20000-60000 | 86 (27.74) | | 28 (35.90) | 17 (22.08) | 18 (23.38) | 23 (29.49) |  |
| >60000 | 18 (5.81) | | 3 (3.85) | 4 (5.19) | 4 (5.19) | 7 (8.97) |  |
| Smoke (%) |  | |  |  |  |  | 0.048 |
| Never | 211 (68.06) | | 50 (64.10) | 49 (63.64) | 53 (68.83) | 59 (75.64) |  |
| Ever | 17 (5.48) | | 2 (2.56) | 4 (5.19) | 3 (3.90) | 8 (10.26) |  |
| Now | 82 (26.45) | | 26 (33.33) | 24 (31.17) | 21 (27.27) | 11 (14.10) |  |
| Seafood (%) |  | |  |  |  |  | 0.583 |
| No | 171 (55.16) | | 43 (55.13) | 45 (58.44) | 40 (51.95) | 43 (55.13) |  |
| Once a day | 1 (0.32) | | 0 (0.00) | 0 (0.00) | 0 (0.00) | 1 (1.28) |  |
| At least once a week | 28 (9.03) | | 7 (8.97) | 4 (5.19) | 6 (7.79) | 11 (14.10) |  |
| At least once a month | 72 (23.23) | | 17 (21.79) | 21 (27.27) | 21 (27.27) | 13 (16.67) |  |
| At least once a year | 38 (12.26) | | 11 (14.10) | 7 (9.09) | 10 (12.99) | 10 (12.82) |  |
| Hypertension (%) |  | |  |  |  |  | 0.319 |
| No | 190 (61.29) | | 47 (60.26) | 41 (53.25) | 50 (64.94) | 52 (66.67) |  |
| Yes | 120 (38.71) | | 31 (39.74) | 36 (46.75) | 27 (35.06) | 26 (33.33) |  |
| eGFR (M (Q₁, Q₃)) | 101.76 (93.24, 112.03) | | 102.16 (90.41,112.12) | 98.97  (89.46,105.86) | 102.47 (96.25,110.24) | 103.12 (97.67,114.30) | 0.046 |
| BMI (M (Q₁, Q₃)) | 24.80 (23.00, 26.80) | | 24.80 (22.70,26.70) | 24.80  (23.60,27.20) | 24.80  (22.20,27.60) | 24.80 (23.22,26.67) | 0.806 |

**Table S5. Baseline characteristics across quartiles of creatinine-corrected urinary DMA in the Zhaodong population**

| **Characteristics** | | **Total**  n=310 | **Q1** (< 25.12 ug/L cr)  n=78 | **Q2** (25.12-42.75 ug/L cr)  n=77 | **Q3** (42.75-65.99 ug/L cr)  n=77 | **Q4** (≥65.99 ug/L cr)  n=78 | ***P*** |
| --- | --- | --- | --- | --- | --- | --- | --- |
| Age (%) | |  |  |  |  |  | 0.405 |
| 18-39 | | 10 (3.23) | 3 (3.85) | 4 (5.19) | 0 (0.00) | 3 (3.85) |  |
| 39-60 | | 129 (41.61) | 32 (41.03) | 36 (46.75) | 32 (41.56) | 29 (37.18) |  |
| >60 | | 171 (55.16) | 43 (55.13) | 37 (48.05) | 45 (58.44) | 46 (58.97) |  |
| Sex (%) | |  |  |  |  |  | 0.022 |
| Female | | 190 (61.29) | 42 (53.85) | 46 (59.74) | 43 (55.84) | 59 (75.64) |  |
| Male | | 120 (38.71) | 36 (46.15) | 31 (40.26) | 34 (44.16) | 19 (24.36) |  |
| Nationality (%) |  | |  |  |  |  | 0.382 |
| Han Chinese | 292 (94.19) | | 76 (97.44) | 72 (93.51) | 73 (94.81) | 71 (91.03) |  |
| Other ethnic minorities | 18 (5.81) | | 2 (2.56) | 5 (6.49) | 4 (5.19) | 7 (8.97) |  |
| Education (%) |  | |  |  |  |  | 0.782 |
| Primary school and below | 207 (66.77) | | 49 (62.82) | 50 (64.94) | 54 (70.13) | 54 (69.23) |  |
| Junior high school | 79 (25.48) | | 21 (26.92) | 23 (29.87) | 18 (23.38) | 17 (21.79) |  |
| High school or above | 24 (7.74) | | 8 (10.26) | 4 (5.19) | 5 (6.49) | 7 (8.97) |  |
| Income (%) |  | |  |  |  |  | 0.070 |
| <20000 | 206 (66.45) | | 47 (60.26) | 54 (70.13) | 59 (76.62) | 46 (58.97) |  |
| 20000-60000 | 86 (27.74) | | 29 (37.18) | 18 (23.38) | 14 (18.18) | 25 (32.05) |  |
| >60000 | 18 (5.81) | | 2 (2.56) | 5 (6.49) | 4 (5.19) | 7 (8.97) |  |
| Smoke (%) |  | |  |  |  |  | 0.032 |
| Never | 211 (68.06) | | 52 (66.67) | 45 (58.44) | 56 (72.73) | 58 (74.36) |  |
| Ever | 17 (5.48) | | 2 (2.56) | 4 (5.19) | 3 (3.90) | 8 (10.26) |  |
| Now | 82 (26.45) | | 24 (30.77) | 28 (36.36) | 18 (23.38) | 12 (15.38) |  |
| Seafood (%) |  | |  |  |  |  | 0.655 |
| No | 171 (55.16) | | 44 (56.41) | 42 (54.55) | 44 (57.14) | 41 (52.56) |  |
| Once a day | 1 (0.32) | | 0 (0.00) | 0 (0.00) | 0 (0.00) | 1 (1.28) |  |
| At least once a week | 28 (9.03) | | 5 (6.41) | 7 (9.09) | 6 (7.79) | 10 (12.82) |  |
| At least once a month | 72 (23.23) | | 20 (25.64) | 22 (28.57) | 16 (20.78) | 14 (17.95) |  |
| At least once a year | 38 (12.26) | | 9 (11.54) | 6 (7.79) | 11 (14.29) | 12 (15.38) |  |
| Hypertension (%) |  | |  |  |  |  | 0.691 |
| No | 190 (61.29) | | 46 (58.97) | 44 (57.14) | 49 (63.64) | 51 (65.38) |  |
| Yes | 120 (38.71) | | 32 (41.03) | 33 (42.86) | 28 (36.36) | 27 (34.62) |  |
| eGFR (M (Q₁, Q₃)) | 101.76 (93.24, 112.03) | | 101.65 (93.48,111.76) | 102.54 (92.28,112.48) | 101.11 (92.54,107.83) | 103.60 (96.81,114.71) | 0.207 |
| BMI (M (Q₁, Q₃)) | 24.80  (23.00, 26.80) | | 24.45 (22.63,26.70) | 24.80  (23.40,26.70) | 24.80  (22.90,27.60) | 24.80 (23.42,27.08) | 0.721 |
