# Supplementary material for: Arsenic species and lipid metabolism under low-to-moderate arsenic exposure: evidence from NHANES and a Chinese adult population
Source: Front Endocrinol (Lausanne). 2026 Aug 18;17:1919240. doi: 10.3389/fendo.2026.1919240 (PMC13529509; doi:10.3389/fendo.2026.1919240)
Supplement: Supplementary file 1 [file DataSheet1.zip › Table S6.docx]

**Table S6. Crude and adjusted logistic associations between quartiles of creatinine-corrected urinary total arsenic and arsenic species and lipid metabolism abnormalities in NHANES and the Zhaodong population**

| **Characteristics** | | **Quartile** | **NHANES, weighted OR (95% CI)** | |  | **Zhaodong population OR (95% CI)** | |
| --- | --- | --- | --- | --- | --- | --- | --- |
|  |  |  | Model 1 | Model 2 |  | Model 1 | Model 2 |
| **TG** | | | | | | | |
|  | **UTA** | Q1 | 1.00 (Reference) | 1.00 (Reference) |  | 1.00 (Reference) | 1.00 (Reference) |
|  |  | Q2 | 1.32 (0.74 ~ 2.37) | 1.49 (0.79 ~ 2.83) |  | 1.26 (0.67 ~ 2.37) | 1.22 (0.61 ~ 2.44) |
|  |  | Q3 | 1.44 (0.82 ~ 2.53) | 1.53 (0.80 ~ 2.91) |  | 1.14 (0.60 ~ 2.14) | 1.23 (0.61 ~ 2.45) |
|  |  | Q4 | 1.00 (0.57 ~ 1.77) | 1.27 (0.64 ~ 2.52) |  | 1.05 (0.56 ~ 1.98) | 1.07 (0.53 ~ 2.18) |
|  | **DMA** | Q1 | 1.00 (Reference) | 1.00 (Reference) |  | 1.00 (Reference) | 1.00 (Reference) |
|  |  | Q2 | 0.80 (0.42 ~ 1.51) | 0.99 (0.47 ~ 2.07) |  | 1.82 (0.96 ~ 3.44) | 1.71 (0.86 ~ 3.42) |
|  |  | Q3 | 1.42 (0.83 ~ 2.44) | 1.37 (0.70 ~ 2.70) |  | 1.20 (0.63 ~ 2.26) | 1.26 (0.63 ~ 2.52) |
|  |  | Q4 | 0.80 (0.44 ~ 1.44) | 0.93 (0.44 ~ 1.98) |  | 1.30 (0.69 ~ 2.44) | 1.19 (0.58 ~ 2.43) |
|  |  |  |  |  |  |  |  |
| **LDL-C** | | | | | | | |
|  | **UTA** | Q1 | 1.00 (Reference) | 1.00 (Reference) |  | 1.00 (Reference) | 1.00 (Reference) |
|  |  | Q2 | 0.83 (0.46 ~ 1.50) | 0.82 (0.45 ~ 1.48) |  | 1.30 (0.59 ~ 2.85) | 1.18 (0.51 ~ 2.72) |
|  |  | Q3 | 0.66 (0.42 ~ 1.05) | 0.76 (0.49 ~ 1.18) |  | 1.60 (0.74 ~ 3.47) | 1.45 (0.64 ~ 3.28) |
|  |  | Q4 | 0.78 (0.46 ~ 1.32) | 0.88 (0.51 ~ 1.53) |  | 0.45 (0.17 ~ 1.19) | 0.33 (0.12 ~ 0.93)*^&^ |
|  | **DMA** | Q1 | 1.00 (Reference) | 1.00 (Reference) |  | 1.00 (Reference) | 1.00 (Reference) |
|  |  | Q2 | 1.19 (0.69 ~ 2.06) | 1.15 (0.64 ~ 2.04) |  | 1.02 (0.47 ~ 2.21) | 0.97 (0.42 ~ 2.23) |
|  |  | Q3 | 0.64 (0.33 ~ 1.24) | 0.62 (0.34 ~ 1.12) |  | 1.27 (0.60 ~ 2.70) | 1.32 (0.59 ~ 2.97) |
|  |  | Q4 | 1.11 (0.71 ~ 1.73) | 1.22 (0.72 ~ 2.06) |  | 0.38 (0.15 ~ 0.99)*^&^ | 0.28 (0.10 ~ 0.79)*^#^ |
|  |  |  |  |  |  |  |  |
| **Non-HDL-C** | | | | | | | |
|  | **UTA** | Q1 | 1.00 (Reference) | 1.00 (Reference) |  | 1.00 (Reference) | 1.00 (Reference) |
|  |  | Q2 | 1.14 (0.80 ~ 1.63) | 1.25 (0.85 ~ 1.85) |  | 1.40 (0.67 ~ 2.96) | 1.41 (0.64 ~ 3.12) |
|  |  | Q3 | 1.02 (0.73 ~ 1.43) | 1.13 (0.80 ~ 1.58) |  | 0.81 (0.41 ~ 1.63) | 0.65 (0.31 ~ 1.37) |
|  |  | Q4 | 0.97 (0.70 ~ 1.33) | 1.20 (0.82 ~ 1.77) |  | 1.14 (0.56 ~ 2.35) | 1.00 (0.45 ~ 2.23) |
|  | **DMA** | Q1 | 1.00 (Reference) | 1.00 (Reference) |  | 1.00 (Reference) | 1.00 (Reference) |
|  |  | Q2 | 0.87 (0.62 ~ 1.22) | 0.81 (0.55 ~ 1.20) |  | 0.98 (0.48 ~ 2.02) | 0.93 (0.44 ~ 2.00) |
|  |  | Q3 | 1.01 (0.74 ~ 1.38) | 0.99 (0.72 ~ 1.35) |  | 0.81 (0.40 ~ 1.64) | 0.72 (0.34 ~ 1.52) |
|  |  | Q4 | 0.69 (0.49 ~ 0.97)*^#^ | 0.76 (0.52 ~ 1.12) |  | 1.24 (0.59 ~ 2.59) | 1.09 (0.48 ~ 2.47) |

Notes: NHANES: Model 1: Unadjusted (Crude); Model 2: Adjust (Sex, Age, Race, Education, PIR, Cotinine, Hypertension, BMI, EGFR, Urinary AsB, Data release number). Zhaodong population: Model 1: Unadjusted (Crude); Model 2: Adjust (Age, Sex, Nationality, Education, Income, BMI, eGFR, Smoke, Seafood, Hypertension). Signif. codes: ‘*’ <0.05. ‘#’ indicates an FDR-adjusted q value <0.1. ‘&’ indicates an FDR-adjusted q value between 0.10 and 0.15, representing suggestive evidence. UTA, urinary total arsenic; DMA, dimethylarsinic acid.
