# Supplementary material for: Arsenic species and lipid metabolism under low-to-moderate arsenic exposure: evidence from NHANES and a Chinese adult population
Source: Front Endocrinol (Lausanne). 2026 Aug 18;17:1919240. doi: 10.3389/fendo.2026.1919240 (PMC13529509; doi:10.3389/fendo.2026.1919240)
Supplement: Supplementary file 1 [file DataSheet1.zip › Table S7-S9.docx]

**Table S7. Baseline characteristics across quartiles of creatinine-corrected urinary MMA in NHANES**

| **Characteristics** | **Total**  N= 52612536  n= 2104 | **Q1**  (<0.27 ug/L cr)  N= 13108648  n= 523 | **Q2**  (0.27-0.50 ug/L cr)  N= 12725468  n= 490 | **Q3**  (0.50-0.91 ug/L cr)  N= 13554992  n= 548 | **Q4**  (**≥** 0.91 ug/L cr)  N= 13223428  n= 543 | ***P*** |
| --- | --- | --- | --- | --- | --- | --- |
|  |  |  |  |  |  |  |
| Sex, n (%) |  |  |  |  |  | <0.001 |
| Female | 857 (37.61) | 245 (43.96) | 217 (43.37) | 223 (35.05) | 172 (28.41) |  |
| Male | 1247 (62.39) | 278 (56.04) | 273 (56.63) | 325 (64.95) | 371 (71.59) |  |
| Age, n (%) |  |  |  |  |  | 0.490 |
| 20-39 | 495 (26.67) | 123 (26.97) | 126 (29.96) | 130 (26.18) | 116 (23.71) |  |
| 40-60 | 766 (41.81) | 190 (40.02) | 179 (39.94) | 213 (45.33) | 184 (41.77) |  |
| > 60 | 843 (31.52) | 210 (33.00) | 185 (30.10) | 205 (28.49) | 243 (34.52) |  |
| Race, n (%) |  |  |  |  |  | <0.001 |
| Mexican American | 271 (5.84) | 61 (5.53) | 69 (6.74) | 73 (6.02) | 68 (5.08) |  |
| Other Hispanic | 197 (3.96) | 37 (2.82) | 32 (3.38) | 53 (4.08) | 75 (5.52) |  |
| Non-Hispanic White | 1106 (77.63) | 254 (76.10) | 253 (76.27) | 300 (77.92) | 299 (80.16) |  |
| Non-Hispanic Black | 359 (7.46) | 131 (10.65) | 101 (8.81) | 84 (7.18) | 43 (3.30) |  |
| Other Race - Including Multi-Racial | 171 (5.11) | 40 (4.90) | 35 (4.80) | 38 (4.80) | 58 (5.94) |  |
| Education, n (%) |  |  |  |  |  | 0.002 |
| Less Than 9th Grade | 205 (5.04) | 34 (3.77) | 42 (4.15) | 65 (6.87) | 64 (5.29) |  |
| 9-11th Grade (Includes 12th grade with no diploma) | 289 (9.33) | 68 (8.29) | 65 (9.23) | 81 (10.46) | 75 (9.32) |  |
| High School Grad/GED or Equivalent | 501 (24.58) | 150 (30.24) | 123 (26.34) | 112 (20.49) | 116 (21.49) |  |
| Some College or AA degree | 686 (33.96) | 193 (40.08) | 159 (31.12) | 170 (30.45) | 164 (34.21) |  |
| College Graduate or above | 423 (27.09) | 78 (17.62) | 101 (29.17) | 120 (31.73) | 124 (29.70) |  |
| PIR, n (%) |  |  |  |  |  | 0.017 |
| ≤1.3 | 716 (22.11) | 170 (21.64) | 159 (20.20) | 184 (23.01) | 203 (23.48) |  |
| 1.3-3.5 | 794 (37.43) | 228 (45.19) | 189 (37.13) | 211 (36.81) | 166 (30.64) |  |
| ＞3.5 | 594 (40.47) | 125 (33.17) | 142 (42.66) | 153 (40.19) | 174 (45.87) |  |
| Cotinine, n (%) |  |  |  |  |  | 0.810 |
| < 0.05 | 1136 (57.00) | 268 (55.57) | 255 (57.74) | 299 (58.13) | 314 (56.53) |  |
| 0.05-10 | 410 (16.53) | 125 (19.79) | 90 (15.53) | 97 (15.27) | 98 (15.54) |  |
| > 10 | 558 (26.48) | 130 (24.64) | 145 (26.73) | 152 (26.60) | 131 (27.93) |  |
| Hypertension, n (%) |  |  |  |  |  | 0.061 |
| No | 939 (51.62) | 207 (44.08) | 230 (53.44) | 250 (53.86) | 252 (55.03) |  |
| Yes | 1165 (48.38) | 316 (55.92) | 260 (46.56) | 298 (46.14) | 291 (44.97) |  |
| Diabetes, n (%) |  |  |  |  |  | 0.387 |
| No | 1558 (79.36) | 377 (75.69) | 374 (80.87) | 412 (79.57) | 395 (81.33) |  |
| Yes | 546 (20.64) | 146 (24.31) | 116 (19.13) | 136 (20.43) | 148 (18.67) |  |
| BMI, Mean (SE) | 29.95 (0.23) | 32.10 (0.39) | 30.74 (0.51) | 28.70 (0.37) | 28.34 (0.36) | <0.001 |
| EGFR, Mean (SE) | 92.47 (0.61) | 91.17 (1.15) | 91.38 (1.30) | 92.68 (1.16) | 94.61 (1.29) | 0.268 |
| Urinary AsB, Mean (SE) | 0.76 (0.01) | 0.82 (0.00) | 0.77 (0.01) | 0.76 (0.01) | 0.71 (0.02) | <0.001 |
| Data release number, n (%) | |  |  |  |  | <0.001 |
| 2009-2010 | 442 (15.51) | 9 (1.26) | 88 (11.92) | 135 (17.24) | 210 (31.30) |  |
| 2011-2012 | 344 (18.35) | 13 (1.97) | 68 (14.39) | 101 (20.75) | 162 (35.92) |  |
| 2013-2014 | 384 (18.00) | 101 (17.79) | 97 (18.99) | 115 (20.31) | 71 (14.90) |  |
| 2015-2016 | 374 (18.97) | 140 (28.56) | 113 (26.22) | 81 (16.53) | 40 (4.99) |  |
| 2017-2020 | 560 (29.17) | 260 (50.41) | 124 (28.48) | 116 (25.17) | 60 (12.88) |  |

**Table S8. Baseline characteristics across quartiles of creatinine-corrected urinary As^III^ in NHANES**

| **Characteristics** | **Total**  N= 52612536  n= 2104 | **Q1**  (<0.13 ug/L cr)  N= 12899261  n= 491 | **Q2**  (0.13-0.31 ug/L cr)  N= 13405747  n= 479 | **Q3**  (0.31-0.69 ug/L cr)  N= 13140533  n= 571 | **Q4**  (**≥**0.69 ug/L cr)  N= 13166995  n= 563 | ***P*** |
| --- | --- | --- | --- | --- | --- | --- |
|  |  |  |  |  |  |  |
| Sex, n (%) |  |  |  |  |  | <0.001 |
| Female | 857 (37.61) | 236 (47.39) | 194 (36.68) | 229 (36.17) | 198 (30.41) |  |
| Male | 1247 (62.39) | 255 (52.61) | 285 (63.32) | 342 (63.83) | 365 (69.59) |  |
| Age, n (%) |  |  |  |  |  | 0.261 |
| 20-39 | 495 (26.67) | 100 (24.89) | 117 (26.87) | 139 (27.07) | 139 (27.81) |  |
| 40-60 | 766 (41.81) | 174 (36.75) | 171 (44.59) | 217 (42.71) | 204 (43.03) |  |
| > 60 | 843 (31.52) | 217 (38.36) | 191 (28.53) | 215 (30.22) | 220 (29.16) |  |
| Race, n (%) |  |  |  |  |  | <0.012 |
| Mexican American | 271 (5.84) | 46 (4.87) | 56 (5.27) | 87 (7.39) | 82 (5.81) |  |
| Other Hispanic | 197 (3.96) | 34 (2.90) | 36 (3.80) | 58 (4.50) | 69 (4.62) |  |
| Non-Hispanic White | 1106 (77.63) | 264 (79.33) | 237 (75.54) | 291 (75.97) | 314 (79.76) |  |
| Non-Hispanic Black | 359 (7.46) | 111 (9.00) | 102 (7.86) | 92 (8.27) | 54 (4.74) |  |
| Other Race - Including Multi-Racial | 171 (5.11) | 36 (3.91) | 48 (7.53) | 43 (3.87) | 44 (5.07) |  |
| Education, n (%) |  |  |  |  |  | 0.162 |
| Less Than 9th Grade | 205 (5.04) | 40 (5.03) | 38 (4.03) | 62 (6.03) | 65 (5.09) |  |
| 9-11th Grade (Includes 12th grade with no diploma) | 289 (9.33) | 60 (8.19) | 52 (8.17) | 93 (11.71) | 84 (9.27) |  |
| High School Grad/GED or Equivalent | 501 (24.58) | 133 (29.33) | 121 (26.73) | 121 (20.99) | 126 (21.34) |  |
| Some College or AA degree | 686 (33.96) | 175 (36.18) | 166 (33.89) | 178 (31.07) | 167 (34.73) |  |
| College Graduate or above | 423 (27.09) | 83 (21.27) | 102 (27.19) | 117 (30.20) | 121 (29.57) |  |
| PIR, n (%) |  |  |  |  |  | 0.038 |
| ≤1.3 | 716 (22.11) | 156 (22.91) | 157 (19.91) | 206 (24.74) | 197 (20.94) |  |
| 1.3-3.5 | 794 (37.43) | 220 (41.88) | 180 (39.45) | 208 (37.14) | 186 (31.30) |  |
| ＞3.5 | 594 (40.47) | 115 (35.22) | 142 (40.64) | 157 (38.12) | 180 (47.76) |  |
| Cotinine, n (%) |  |  |  |  |  | 0.997 |
| < 0.05 | 1136 (57.00) | 256 (57.69) | 255 (57.34) | 305 (55.60) | 320 (57.36) |  |
| 0.05-10 | 410 (16.53) | 105 (16.67) | 92 (16.82) | 110 (16.90) | 103 (15.71) |  |
| > 10 | 558 (26.48) | 130 (25.64) | 132 (25.84) | 156 (27.50) | 140 (26.92) |  |
| Hypertension, n (%) |  |  |  |  |  | 0.102 |
| No | 939 (51.62) | 195 (47.68) | 203 (49.32) | 264 (52.10) | 277 (57.33) |  |
| Yes | 1165 (48.38) | 296 (52.32) | 276 (50.68) | 307 (47.90) | 286 (42.67) |  |
| Diabetes, n (%) |  |  |  |  |  | 0.097 |
| No | 1558 (79.36) | 344 (74.09) | 362 (80.55) | 436 (80.86) | 416 (81.81) |  |
| Yes | 546 (20.64) | 147 (25.91) | 117 (19.45) | 135 (19.14) | 147 (18.19) |  |
| BMI, Mean (SE) | 29.95 (0.23) | 31.29 (0.38) | 30.27 (0.47) | 29.96 (0.46) | 28.31 (0.39) | <0.001 |
| EGFR, Mean (SE) | 92.47 (0.61) | 88.10 (1.11) | 90.89 (1.32) | 94.48 (1.14) | 96.36 (1.32) | <0.001 |
| Urinary AsB, Mean (SE) | 0.76 (0.01) | 0.82 (0.00) | 0.82 (0.00) | 0.76 (0.01) | 0.66 (0.02) | <0.001 |
| Data release number, n (%) | |  |  |  |  | <0.001 |
| 2009-2010 | 442 (15.51) | 0 (0.00) | 13 (1.89) | 135 (17.42) | 294 (42.66) |  |
| 2011-2012 | 344 (18.35) | 9 (1.73) | 88 (17.53) | 135 (27.27) | 112 (26.53) |  |
| 2013-2014 | 384 (18.00) | 78 (15.22) | 104 (19.73) | 117 (20.24) | 85 (16.74) |  |
| 2015-2016 | 374 (18.97) | 128 (28.49) | 114 (25.20) | 94 (16.09) | 38 (6.18) |  |
| 2017-2020 | 560 (29.17) | 276 (54.56) | 160 (35.65) | 90 (18.98) | 34 (7.89) |  |

**Table S9. Baseline characteristics across quartiles of creatinine-corrected urinary As^V^ in NHANES**

| **Characteristics** | **Total**  N= 52612536  n= 2104 | **Q1**  (<0.43 ug/L cr)  N= 13139299  n= 526 | **Q2**  (0.43-0.74 ug/L cr)  N= 12981381  n= 600 | **Q3**  (0.74-1.33 ug/L cr)  N= 13240589  n= 497 | **Q4**  (**≥**1.33 ug/L cr)  N= 13251267  n= 481 | ***P*** |
| --- | --- | --- | --- | --- | --- | --- |
|  |  |  |  |  |  |  |
| Sex, n (%) |  |  |  |  |  | <0.001 |
| Female | 857 (37.61) | 278 (51.80) | 286 (44.58) | 172 (30.67) | 121 (23.65) |  |
| Male | 1247 (62.39) | 248 (48.20) | 314 (55.42) | 325 (69.33) | 360 (76.35) |  |
| Age, n (%) |  |  |  |  |  | 0.005 |
| 20-39 | 495 (26.67) | 166 (36.27) | 124 (23.12) | 104 (22.63) | 101 (24.66) |  |
| 40-60 | 766 (41.81) | 182 (37.47) | 222 (41.14) | 194 (47.33) | 168 (41.25) |  |
| > 60 | 843 (31.52) | 178 (26.26) | 254 (35.74) | 199 (30.04) | 212 (34.09) |  |
| Race, n (%) |  |  |  |  |  | <0.001 |
| Mexican American | 271 (5.84) | 68 (7.21) | 82 (6.63) | 62 (4.72) | 59 (4.82) |  |
| Other Hispanic | 197 (3.96) | 42 (3.47) | 62 (4.61) | 51 (4.19) | 42 (3.58) |  |
| Non-Hispanic White | 1106 (77.63) | 258 (74.81) | 285 (73.99) | 279 (79.60) | 284 (82.02) |  |
| Non-Hispanic Black | 359 (7.46) | 126 (10.44) | 122 (10.01) | 65 (5.62) | 46 (3.85) |  |
| Other Race - Including Multi-Racial | 171 (5.11) | 32 (4.07) | 49 (4.76) | 40 (5.88) | 50 (5.72) |  |
| Education, n (%) |  |  |  |  |  | 0.006 |
| Less Than 9th Grade | 205 (5.04) | 36 (4.34) | 74 (5.89) | 53 (5.67) | 42 (4.28) |  |
| 9-11th Grade (Includes 12th grade with no diploma) | 289 (9.33) | 81 (9.76) | 93 (13.07) | 59 (7.81) | 56 (6.78) |  |
| High School Grad/GED or Equivalent | 501 (24.58) | 140 (29.04) | 151 (27.10) | 113 (23.72) | 97 (18.56) |  |
| Some College or AA degree | 686 (33.96) | 177 (33.59) | 179 (31.86) | 163 (33.43) | 167 (36.90) |  |
| College Graduate or above | 423 (27.09) | 92 (23.27) | 103 (22.09) | 109 (29.37) | 119 (33.48) |  |
| PIR, n (%) |  |  |  |  |  | 0.011 |
| ≤1.3 | 716 (22.11) | 174 (21.82) | 225 (26.51) | 173 (20.68) | 144 (19.51) |  |
| 1.3-3.5 | 794 (37.43) | 218 (42.55) | 229 (37.99) | 174 (37.83) | 173 (31.39) |  |
| ＞3.5 | 594 (40.47) | 134 (35.63) | 146 (35.50) | 150 (41.49) | 164 (49.10) |  |
| Cotinine, n (%) |  |  |  |  |  | 0.009 |
| < 0.05 | 1136 (57.00) | 242 (48.32) | 324 (55.64) | 272 (60.01) | 298 (63.92) |  |
| 0.05-10 | 410 (16.53) | 118 (20.33) | 119 (16.79) | 91 (14.41) | 82 (14.61) |  |
| > 10 | 558 (26.48) | 166 (31.35) | 157 (27.57) | 134 (25.58) | 101 (21.47) |  |
| Hypertension, n (%) |  |  |  |  |  | 0.752 |
| No | 939 (51.62) | 242 (52.02) | 253 (48.78) | 221 (51.75) | 223 (53.86) |  |
| Yes | 1165 (48.38) | 284 (47.98) | 347 (51.22) | 276 (48.25) | 258 (46.14) |  |
| Diabetes, n (%) |  |  |  |  |  | 0.406 |
| No | 1558 (79.36) | 404 (82.29) | 421 (75.70) | 369 (79.54) | 364 (79.86) |  |
| Yes | 546 (20.64) | 122 (17.71) | 179 (24.30) | 128 (20.46) | 117 (20.14) |  |
| BMI, Mean (SE) | 29.95 (0.23) | 31.56 (0.36) | 30.04 (0.34) | 30.11 (0.50) | 28.10 (0.40) | <0.001 |
| EGFR, Mean (SE) | 92.47 (0.61) | 92.52 (1.05) | 89.43 (1.20) | 93.01 (1.19) | 94.86 (1.32) | <0.020 |
| Urinary AsB, Mean (SE) | 0.76 (0.01) | 0.79 (0.01) | 0.76 (0.01) | 0.76 (0.01) | 0.75 (0.01) | 0.008 |
| Data release number, n (%) | |  |  |  |  | 0.024 |
| 2009-2010 | 442 (15.51) | 71 (9.92) | 138 (16.96) | 111 (16.41) | 122 (18.73) |  |
| 2011-2012 | 344 (18.35) | 74 (14.89) | 91 (18.87) | 91 (18.36) | 88 (21.24) |  |
| 2013-2014 | 384 (18.00) | 110 (19.79) | 109 (19.22) | 85 (16.30) | 80 (16.74) |  |
| 2015-2016 | 374 (18.97) | 109 (23.98) | 100 (14.76) | 87 (19.25) | 78 (17.85) |  |
| 2017-2020 | 560 (29.17) | 162 (31.42) | 162 (30.19) | 123 (29.68) | 113 (25.44) |  |
